# Lasamide Containing Sulfonylpiperazines as Effective Agents for the Management of Glaucoma Associated Symptoms

**DOI:** 10.1002/cmdc.202400601

**Published:** 2024-11-08

**Authors:** Jaydeo T. Kilbile, Suryakant B. Sapkal, Gioele Renzi, Ilaria D'Agostino, Mohamed Boudjelal, Yasinalli Tamboli, Luigi Cutarella, Mattia Mori, Silvia Sgambellone, Serafina Villano, Silvia Marri, Laura Lucarini, Simone Carradori, Fabrizio Carta, Claudiu T. Supuran

**Affiliations:** ^1^ Department of Chemistry School of Basic and Applied Sciences MGM University Chhatrapati Sambhajinagar 431003 MS India; ^2^ NEUROFARBA Department Sezione di Scienze Farmaceutiche e Nutraceutiche University of Florence 50019 Sesto Fiorentino, Florence Italy; ^3^ Department of Pharmacy University of Pisa 56126 Pisa Italy; ^4^ King Abdullah International Medical Research Center (KAIMRC) King Saud Bin Abdulaziz University for Health Sciences Ministry of National Guard-Health Affairs Riyadh 14811 Saudi Arabia; ^5^ Department of Biotechnology Chemistry and Pharmacy University of Siena via Aldo Moro 2 53100 Siena Italy; ^6^ NEUROFARBA Department Section of Pharmacology and Toxicology University of Florence 50139 Florence Italy; ^7^ Department of Pharmacy University “G. d'Annunzio” Chieti-Pescara 66100 Chieti Italy

**Keywords:** Lasamide, Sulfonylpiperazines, Glaucoma, IOP, Carbonic Anhydrase

## Abstract

A series of 2,4‐dichloro‐5‐{[4‐(phenylsulfonyl)piperazin‐1‐yl]carbonyl}benzenesulfonamides were designed and synthesized through amidation of Lasamide **1** with substituted piperazines. The newly obtained compounds demonstrated remarkable inhibition potency and selectivity for the human (h) expressed Carbonic Anhydrase (CA; EC 4.2.1.1) II isoform. Selected compounds **7** and **9** were investigated in an *in vivo* model of glaucoma and showed relevant performances, with the latter being able to last the effect up to 4 hours. The results herein reported are in sustainment of Lasamide derivatives as a new class of compounds potentially exploitable for the management of uncontrolled intra ocular pressure (IOP).

## Graphical abstract

Amidation of **Lasamide** with piperazines led to potent and selective inhibitors of the human Carbonic Anhydrase (hCA, EC: 4.2.1.1) II isoform, whose role in glaucoma has been longtime validated. Insight into the interaction with the enzyme was gained through *in silico* studies. Two compounds (**7** and **9**) were demonstrated to be efficient in lowering the uncontrolled intraocular pressure in *in vivo* glaucoma model in rabbits.

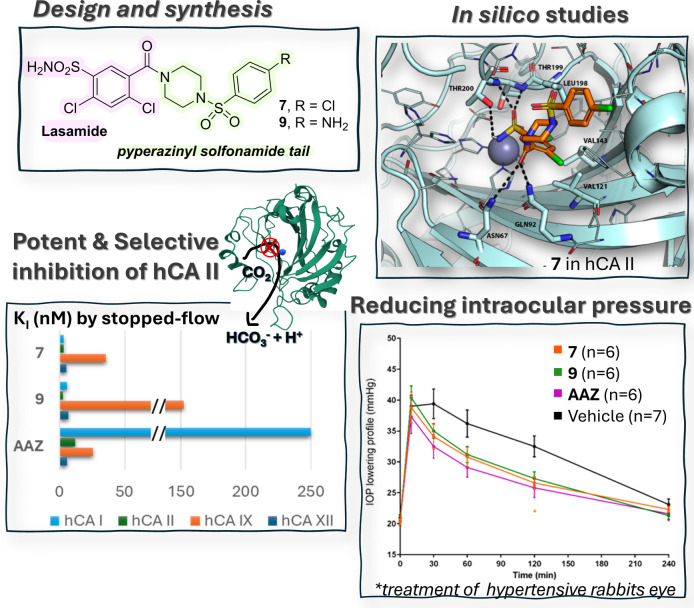

## Introduction

**Figure 1:**
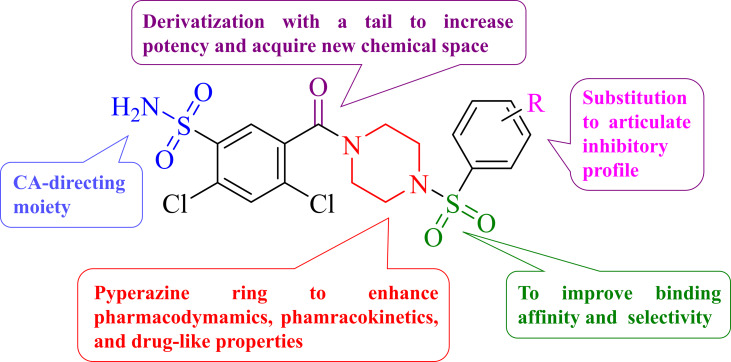
Drug design strategy for sulfonylpiperazino‐Lasamide hybrids herein reported.

Pharmacological treatment of glaucoma is mainly focused on lowering the intraocular pressure (IOP) in order to prevent any irreversible damage to the optic nerves.[^1^, ^2^, ^3^] Recent advancements and approaches include novel drug classes, improvements in drug delivery systems and setting of combination therapies.^[4]^ Well established drugs account for diverse classes among which the most effectives are: *i*) analogues of prostaglandins^[5]^; *ii*) Rho kinase inhibitors[^6^, ^7^]; *iii*) Nitric Oxide donors^[8]^ *iv*) Adenosine receptor agonists^[9]^ and *v*) inhibitors of the Carbonic Anhydrase (CA; EC 4.2.1.1) enzymes.^[10]^ The latter were the first drugs approved for such purposes and currently are extensively used for the management of IOP, also in consideration of the high safety profiles of Dorzolamide (TRUSOPT©) and Brinzolamide (AZOPT©) when administered topically.^[11]^ Despite such advantages, the search for new chemical entities worth developing for the obtainment of new CA inhibitors (CAIs) still thrives, also in consideration of intellectual property and marketing protection strategies. In this context, we envisaged to make use of the prototypic CAI primary sulfonamide moiety contained in the well‐known drug Lasamide **1** and to manipulate its carboxylic group as schematically reported in Figure 1, also inspired by our recent contribution.^[12]^

Specifically, we aimed at developing new molecules by incorporating the piperazine group since it is established as successful and versatile scaffold in Medicinal Chemistry being beneficial in endowing drugs with adequate pharmacokinetic and pharmacodynamic properties[^13^, ^14^] Additionally, sulfonylpiperazine congeners serve as specialized scaffolds with distinct biological effects^[15]^ and enhancement of binding affinity of the ligands towards the targets of interest.^[16]^ Finally, the terminal aryl moiety was intended as a platform to introduce chemical moieties able to specifically interact with unique aminoacids defining the rim of each hCAs catalytic cleft and therefore contribute to endowing isoform selectivity to the ligands.^[17]^

## Results and Discussion

### Chemistry

**Scheme 1:**
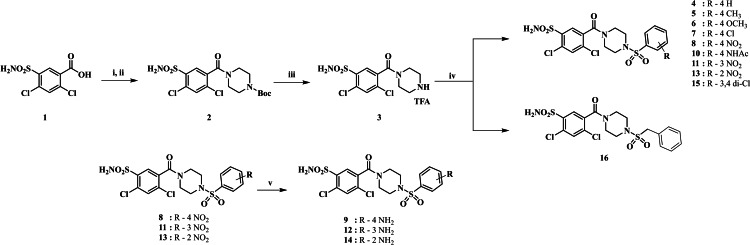
Synthetic pathway for sulfonylpiperazino derivatives **4**–**16**; Reagents and conditions: *i*) SOCl_2_, toluene, 45 °C, 6 h; *ii*) *N*‐Boc piperazine, TEA, THF, 5 °C to rt, 5 h; *iii*) TFA, DCM, 5 °C to rt, 2 h; *iv*) appropriate sulfonyl chloride, K_2_CO_3_, DMF, r.t., 3 h; *v*) Fe powder/aq. HCl, EtOH/H_2_O (1/1), 75 °C, 3 h.

A series of twelve novel sulfonylpiperazino derivatives were synthesized from the commercially available Lasamide **1** and variegate arylsulfonyl chlorides. At first, we attempted the amidation of **1** with *tert*‐butyl piperazine‐1‐carboxylate (*N*‐Boc‐piperazine) to afford *tert*‐butyl 4‐[(2,4‐dichloro‐5‐sulfamoylphenyl)carbonyl]piperazine‐1‐carboxylate **2** in low yields by means of long‐established coupling agents such as 1,1′‐carbonyldiimidazole (CDI) or 1‐ethyl‐3‐(3‐dimethylaminopropyl)carbodiimide/1‐hydroxybenzotriazole (EDC/HOBt).^[18]^ Better results were obtained with hexafluorophosphate azabenzotriazole tetramethyl uronium (HATU) and hexafluorophosphate benzotriazole tetramethyl uronium (HBTU) coupling agents although the associated high costs discourages us to make their use for scale up applications. Thus, we conveniently obtained the lasamide derivative **2** by acylation of **1** with thionyl chloride^[19]^ (SOCl_2_) followed by amidation under basic conditions with triethylamine (TEA) in tetrahydrofuran (THF) as solvent. In the next step, Boc derivative **2** was converted to the amine intermediate **3** as trifluoroacetate salt by deprotection using trifluoroacetic acid (TFA) in dichloromethane (DCM) at room temperature (r.t.). Further, the reaction of **3** with substituted (*ortho*, *meta*, and *para*) sulfonyl chlorides afforded derivatives **4**–**8**, **10**, **11**, **13**, **15** and **16**. Reduction of the nitro moiety in **8**, **11** and **13** was performed with iron (Fe) powder/aq. HCl in a mixture of ethanol/water (EtOH/H_2_O 1/1) at 75 °C to afford the corresponding anilines **9**, **12** and **14** respectively (Scheme 1).

All the final products were isolated through solvent extraction methods using ethyl acetate followed by recrystallization from diisopropyl ether or a suitable mixture of isopropyl alcohol (IPA) and diisopropyl ether. Derivatives **9**, **12** and **14** were purified by silica gel column chromatography eluting with 10 % methanol in dichloromethane (10 % MeOH/DCM).

The structures of synthesized compounds were elucidated by means of ^1^H NMR, ^13^C NMR, ESI‐MS and elemental analyses and were >96 % purity.

### *In Vitro* Enzymatic Assessment on hCAs

The inhibition profiles of Lasamide **1**, intermediate **2**, the synthetic sulfonylpiperazines **4**–**16** on the physiologically relevant hCAs I, II, IX and XII were determined through the stopped‐flow CO_2_ hydrase assay^[20]^ and were compared to the commercially available acetazolamide (**AAZ**) compounds as references. Inhibition constants (*K_I_*) and selectivity indexes for isoform of interest (SI _IX/II_=*K*_I hCA IX_/*K*_I hCA II_) were reported in Table 1.

**Table 1:** Inhibition data of Lasamide **1**, intermediate **2**, derivatives **4**–**16** and the references **AAZ** on hCAs I, II, IX and XII isoforms.^[20]^ Calculated Selectivity Indexes for the hCA II over the IX isoform (SI _IX/II_=*K*_I hCA IX_/*K*_I hCA II_) are reported.

*K*_I_ (nM)^[*]^					
Compound	hCA I	hCA II	hCA IX	hCA XII	SI _IX/II_
**1**	0.52	0.33	2.6	7.5	7.8
**2**	85.7	3.9	40.1	50.1	–
**4**	9.4	7.4	39.7	7.1	5.36
**5**	64.0	29.0	340.1	62.4	11.72
**6**	9.1	2.8	40.6	11.6	14.5
**7**	8.2	3.4	35.8	5.2	10.52
**8**	79.9	71.2	39.4	7.9	0.55
**9**	5.7	2.6	152.2	6.9	58.53
**10**	8.0	5.4	40.4	7.2	7.50
**11**	6.2	6.6	37.7	7.8	5.70
**12**	5.8	0.8	26.3	5.4	32.87
**13**	7.8	0.3	28.9	7.2	96.33
**14**	5.4	2.8	11.4	9.6	4.07
**15**	7.1	5.7	39.0	9.1	6.84
**16**	0.54	0.07	273.0	0.87	>3000
AAZ	250.0	12.1	25.8	5.7	–

[*] Mean from 3 different assays by a stopped‐flow technique. Errors were in the range of ±5–10 % of the reported values.

All compounds considered in this study were effective nanomolar inhibitors for the tested hCA isoforms with preferential affinity for the isoform II. Detailed structure activity‐relationships (SARs) are reported below for each isoform:

*i*) As for the hCA I, the compounds showed *K*_I_ values higher when compared to the parent Lasamide **1** with the only exception of the benzylsulfonyl **16** which was equipotent (i. e. *K*_I_s of 0.52 and 0.54 nM for **1** and **16** respectively). Among the phenyl derivatives **4**–**15** the kinetic trend was strongly influenced from the moieties installed. For instance, the tolyl **5** heavily spoiled the inhibition potency up to 64.0 nM thus 6.8‐fold less effective when compared to the unsubstituted **4** (i. e. *K*_I_ of 9.4 nM). Introduction at the same position of the methoxy group allowed to regain potency (i. e. *K*_I_ of 9.1 nM for **6**) which was further increased when the chlorine atom was introduced instead (i. e. *K*_I_ of 8.2 nM for **7**) or double substitution occurred (i. e. *K*_I_ of 7.1 nM for **15**). Remarkable regioisomeric effects were observed among the nitro (−NO_2_) containing derivatives **8**, **11** and **13** (Table 1). Specifically, the *para*‐ substitution disfavoured the ligand interaction (i. e. *K*_I_ of 79.9 nM) which was regained when the −NO_2_ moiety was placed in *ortho*‐ (i. e. **13**) and *meta*‐position (i. e. **11**) instead (i. e. *K*_I_s of 6.2 and 7.8 nM for **11** and **13** respectively). Interestingly, reduction of the −NO_2_ groups of **8**, **11** and **13** to afford the corresponding anilino derivatives **9**, **12** and **14** strongly reduced the associated *K*_I_ values and flattened the kinetic trend (i. e. *K*_I_s of 5.7, 5.8 and 5.4 nM for **9**, **12** and **14** respectively). In addition, acetylation of **9** to afford **10** slightly increased the *K*_I_ value up to 8.0 nM (Table 1).

*ii*) The catalytically fast hCA II isoform was much more susceptible to inhibition by the listed compounds when compared to its ubiquitous hCA I counterpart, as most of the *K*_I_ values in Table 1 were within the low nanomolar range. Again, the substitutions at the phenyl moiety in **4**–**10** have major effects on the kinetic trend. For instance, the tolyl **5** was 3.9‐fold less effective than its unsubtituted **4** (i. e. *K*_I_s of 7.4 and 29.0 nM for **4** and **5** respectively). Noteworthy, the introduction at position 4 of the phenyl ring in derivative **4** of moieties endowed with predominant mesomeric effects over the electronic ones, determined enhancement of the inhibition values. The *K*_I_s of compounds **6** (−OCH_3_), **7** (−Cl), **9** (−NH_2_) and **10** (−NHAc) were 2.8, 3.4, 2.6 and 5.4 nM respectively. Again a strong regioisomeric effect was observed among the nitro containing compounds **8**, **11** and **13**, with *K*_I_ values of 71.2 nM for the *para*‐substituted **8**, followed by the *meta*‐ and the *ortho*‐regioisomers (i. e. *K*_I_s of 6.6 and 0.3 nM for the **11** and **13** respectively). **13** Is the most potent phenyl derivative able to inhibit the hCA II with the same potency as the precursor Lasamide **1** (i. e. *K*_I_s of 0.3 nM). The same effect, although with diverse isomeric sequence, was observed among the aniline derivatives **9**, **12** and **14**. In this case the *meta*‐substituted **12** was by far the most effective inhibitor (i. e. *K*_I_ of 0.8 nM) over **9** and **14** which showed close matching *K*_I_ values (i. e. *K*_I_s of 2.6 and 2.8 nM for **9** and **14** respectively). Interestingly, the *N*‐acetylated **10** and the 3,4‐dichlorosubstituted **15** were almost equipotent hCA II inhibitors (i. e. *K*_I_s of 5.4 and 5.7 nM for **10** and **15** respectively). The benzylic derivative **16** was the most potent hCA II and showed a *K*_I_ value of 0.07 nM, thus 4.3‐fold more effective than the parent Lasamide **1** and the *meta*‐substituted aniline **12** (i. e. *K*_I_s of 0.3 nM).

*iii*) A general overlook at the hCA IX inhibition data clearly showed this isoform to be inhibited from the synthesized **4**–**16** at medium nanomolar concentrations, whereas the Lasamide **1** was far more effective with a *K*_I_ value of 2.6 nM (Table 1). Despite the structural differences among **4**, **6**, **8**, **10**, **11**, **15** and the intermediate **2**, their associated inhibition values were all comprised between 37.7 and 40.6 nM, thus not allowing to extract proper SARs for this isoenzyme (Table 1). Clear regioisomeric effect was reported among the aniline derivatives **9**, **12** and **14** which showed *K*_I_ values of 152.2, 26.3 and 11.4 nM respectively, whereas for their nitro counterparts **8**, **11** and **13** the same kinetic trend was retained although highly reduced (i. e. *K*_I_s of 39.4, 37.7 and 28.9 nM for **8**, **11** and **13** respectively). The tolyl derivative **5** was the least effective hCA IX inhibitor among all compounds considered in this study (i. e. *K*_I_ of 340.1 nM). Substitution in 5 of the methyl with a chloro atom instead (i. e. compound **7**) determined a significative increase of the inhibition potency (i. e. *K*_I_ values of 35.8 nM). Finally the benzyl **16** showed inhibition at high concentration (i. e. *K*_I_ of 273.0 nM).

*iv*) Better results in terms of inhibition potency were obtained for the second tumor associated isoform hCA XII although in this case the regioisomeric effects on the kinetics, as previously discussed for the hCAs I, II and IX, were suppressed. For instance, among the NO_2_‐derivatives **8**, **11** and **13** the *K*_I_ values were 7.9, 7.8 and 7.2 nM respectively and thus were equipotent to Lasamide **1** (i. e. *K*_I_ of 7.5 nM; Table 1). Slightly marked *K*_I_ differences were reported for the aniline counterpart **9**, **12** and **14** with *K*_I_s of 6.9, 5.4 and 9.6 nM respectively, thus with the *meta*‐substituted **12** being the most effective hCA XII inhibitor. Monoacetylation of **9** to afford **10** didn't significantly affect the kinetic trend (i. e. *K*_I_s of 6.9 and 7.2 nM for **9** and **10** respectively). Similarly to the hCAs I, II and IX, also in this case substitutions at position 4 of the phenyl tail in compound **4** to afford derivatives **5**–**7** heavily affected the kinetic trend. For instance the tolyl derivative **5** was 8.8‐fold less effective hCA XII inhibitor (i. e. *K*_I_s of 7.1 and 62.4 nM for **4** and **5** respectively). The methoxy moiety in **6** was beneficial for the inhibition potency as gave a *K*_I_ value of 11.6 nM, and even more the insertion at the same position of the chloro atom as in compound **7** further reduced the *K*_I_ value up to 5.2 nM. Introduction of an additional chloro atom to afford the 3,4‐dichloro derivative **15** spoiled the inhibition potency of 1.8‐fold (*K*_I_ value of 9.1 nM). The benzyl **16** was the most effective hCA XII inhibitor among the entire series with a *K*_I_ value of 0.87 nM (Table 1).

### *In vivo* Intraocular Pressure (IOP) Lowering Activity

On the basis of the data reported in Table 1, we considered the best performing compounds towards the hCA isoform expressed in the human eyes and implicated the production of humor aqueous. Specifically, we considered the hCA II as preferential whereas the hCA IX was assumed as off‐target.[^21^, ^22^] During the selection process, we focused on those compounds which contained biochemically stable moieties and adequate solubility features for being considered in *in vivo* experiments and derivatives **7** and **9** fully accomplished to such requirements. The hypotensive effect of selected compounds was assessed in hypertensive rabbits and compared to the reference drug **AAZ** topically administered as eye drops at 1 % w/v concentration. Before administration the animals were injected with 50 μL of hypertonic saline solution into the vitreous of both eyes to induce ocular hypertension, and a vehicle solution (0.9 % NaCl and 0.1 % DMSO in sterile H_2_O) was used as control.

**Figure 2:**
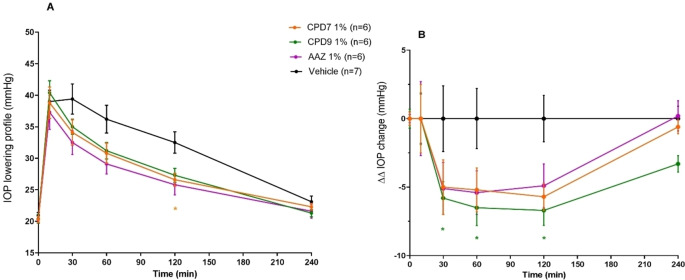
**A**) IOP lowering profile (mmHg) and **B**) ΔΔIOP (mmHg) *vs* time (min) in eyes of hypertensive rabbits treated with 30 μL of 1 % w/v aqueous solution of compounds **7**, **9** and **AAZ** as reference drug. Data are expressed as mean±SEM (n=6/7 eyes); *p<0.05 **7** and **9** *vs* vehicle. Two‐way ANOVA followed by Bonferroni post hoc test.

Overall, data in Figures 2A and B showed that both compounds had the ability to lower the IOP as early as 30 minutes, with a peak effect at 120 minutes after administration, thus longer than the reference **AAZ** which peaked at 60 minutes. In particular the chloro derivative **7** showed a profile superimposable to **AAZ** up to 60 minutes and maintained slightly better performances at 120 followed by a decay rate equal to the reference (Figure 2B). As for the aniline derivative **9**, stronger IOP lowering effect was observed when compared to **7** and to **AAZ** at 30 minutes after administration. The maximum effect was obtained at 60 minutes and was maintained almost constant up to 120 followed by a decrease rate which was far less stiffer than **7** and thus endowed the compound to retain significant activity up to the end of the experimental time frame of 240 minutes (Figure 2B).

### *In silico* Binding Mode Assessment

**Figure 3:**
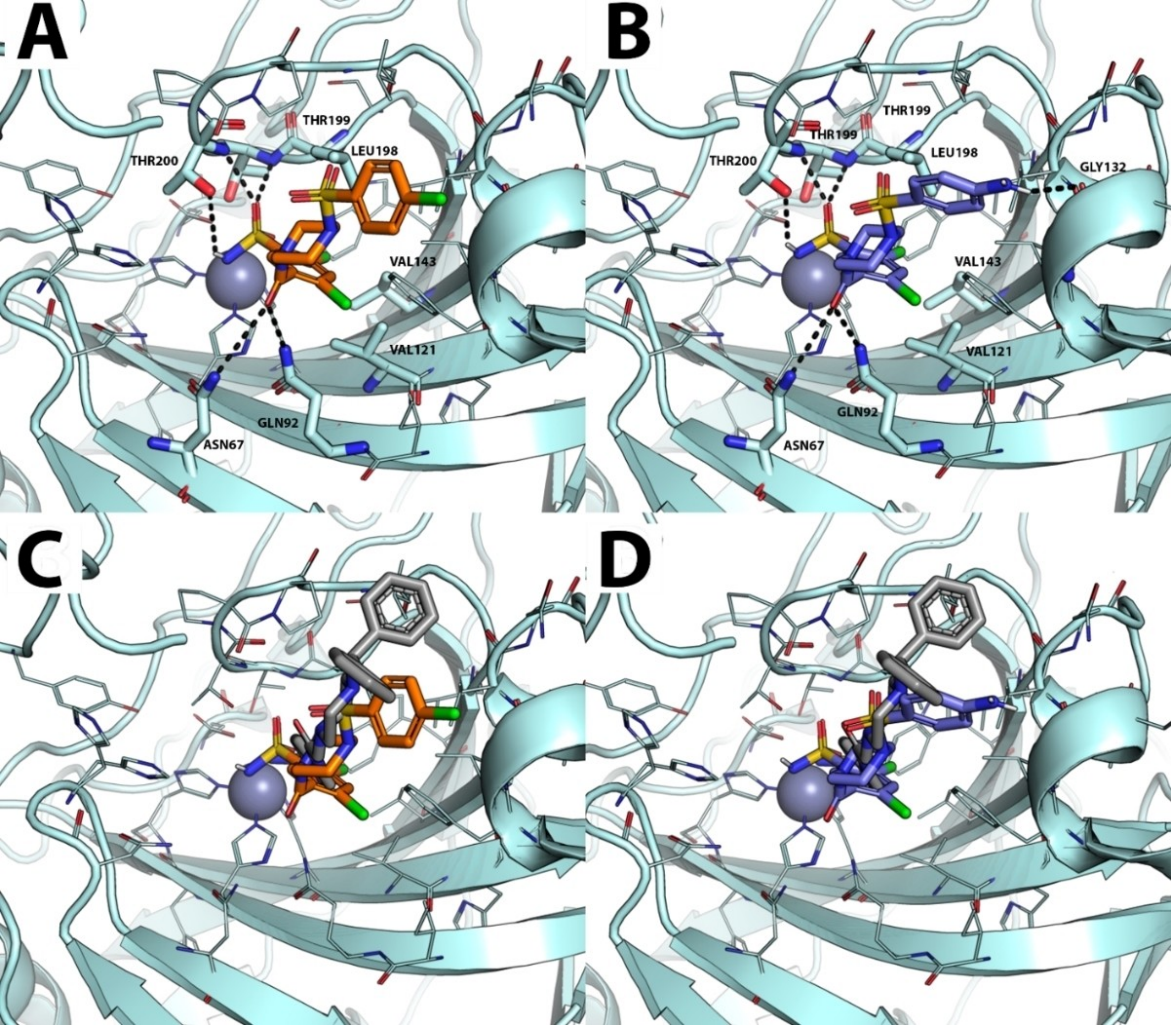
Predicted binding mode of compound **7** and **9** against the crystallographic structure of hCA II (PDB‐ID: 6ZR8, palecyan cartoons and lines). **A**) docking pose of **7**, the molecule is shown as orange sticks; **B**) docking pose of **9**, the molecule is shown as blue sticks. The catalytic Zn(II) ion is shown as a grey sphere. Polar interactions in panels **A** and **B** are highlighted by black dashed lines. In panel **C** and **D**, the overlap between the docking pose of compound **7** and **9** and the co‐crystallized ligand (grey sticks) is shown.

The binding mode and affinity score of selected compounds **7** and **9** were investigated by molecular docking simulations on the hCA II isoform, which is strongly inhibited by the compounds. The X‐ray crystallography structure of hCA II in complex with a sulfonamide inhibitor was selected as a receptor in molecular docking simulations.^[23]^ The reliability of the docking protocol was preliminarily assessed by re‐docking the co‐crystallized inhibitor to hCA II, obtaining a docking pose that overlaps with the crystallographic pose (RMSD <1.0 Å, data not shown). Docking results clearly showed that **7** and **9** bind with a highly superimposable pose. The primary sulfonamide moiety coordinates the catalytic Zn(II) ion with a geometry that corresponds to that of the co‐crystallized ligand (Figures 3C and D). Furthermore, both molecules established H‐bonds with THR‐200, THR‐199, GLN‐92, ASN‐67, while the 1,3‐dichlorophenyl ring docks into a hydrophobic pocket composed of VAL‐143, VAL‐121 and LEU‐141 (Figures 3A and B).

Compound **9** establishes an additional H‐bond with the carbonyl backbone of GLY‐132, which can be replaced by a potential halogen bond in **7**, although this latter interaction type is poorly accounted for in docking simulations. Overall, molecular docking simulations provided a structural hypothesis of the interaction between **7** and **9** with hCA II, suggesting that the sulfonamide group is responsible for Zn(II) coordination, while the tail of the molecule interacts with key residues within the catalytic site, thus corroborating the strong inhibitory activity observed by experiments.

## Conclusions

In conclusion, 13 compounds based on the Lasamide scaffold and containing substituted aryl piperazine tails were designed, synthesized and assessed for their inhibitory activity on the hCAs I, II, IX and XII. The newly synthesized molecules exhibited remarkable inhibition properties for the hCA II isoform with *K*_I_ values ranging from 0.07 to 7.4 nM thus far lower when compared to the reference **AAZ** (*K_I_* of 12.1 nM) and with SIs in disfavour of the hCA IX. Among the series reported, compounds **7** and **9** fulfilled the kinetic profile and solubility requirements necessary to be considered in *in vivo* model of IOP. The results obtained clearly sustained the derivative **9** being far more effective and long lasting than the reference drug **AAZ** in lowering the IOP. *In silico* experiments revealed **7** and **9** can bind within the hCA II catalytic cleft in agreement with the prototypic primary sulfonamides reported in the literature^[24]^ and were superimposable to each other. Overall the achievements reported in this study sustain that manipulation of the known drug Lasamide **1** may give access to a new class of molecules potentially exploitable for the management of uncontrolled IOP.

## Experimental Section

### General Chemistry

All the reagents and anhydrous solvents were purchased from Sigma‐Aldrich, Alfa Aesar, and TCI and used directly without any purification. Nuclear magnetic resonance spectra ^1^H NMR and ^13^C NMR were recorded in DMSO‐*d_6_* using a Bruker Ultrashield NMR spectrometer (Bruker CO., Switzerland) at 700 MHz and 176 MHz, respectively. The chemical shift values (δ) were reported in parts per million (ppm), and splitting patterns are designated as follows: s, singlet; d, doublet; t, triplet; q, quadruplet; m, multiplet; brs, broad singlet; dd, doublet of doublet, and the coupling constant (*J*) is expressed in Hertz (Hz). Thin‐layer chromatography was carried out on silica gel F‐254 plates purchased from Merck with visualization of components by UV light (254 nm). The IR spectra were recorded with an FT‐IR spectrophotometer (Perkin‐Elmer FTIR‐4200). Elemental analyses were analyzed by PerkinElmer Series II 2400 CHNS/O Analyzer. Agilent 6320 Ion Trap mass spectrometer was used to analyze mass spectra (MS).

### Synthetic Procedures

#### Tert‐butyl 4‐[(2,4‐dichloro‐5‐sulfamoylphenyl)carbonyl]piperazine‐1‐carboxylate (2)

To a stirred solution of 2,4‐dichloro‐5‐sulfamoylbenzoic acid (**1**) (40 g, 0.148 mol) in toluene (200 mL), SOCl_2_ (35.2 g, 0.296 mol) was added slowly at 10–15 °C. The reaction mixture was heated to 45 °C and stirred for 6 h. After completion of the reaction, the reaction mass was evaporated under reduced pressure at 45 °C and stripped with cyclohexane (50 mlx2 times) to afford corresponding acyl chloride of compound **1** as a pale yellowish solid (44.5 g). The solution of obtained acyl chloride and THF (240 mL) was cooled to 0–10 °C and TEA (51.65 mL, 0.370 mol) was added. After 15 min stirring, *tert*‐Butyl piperazine‐1‐carboxylate (33.1 g, 0.177 mol) was added, and the mixture was stirred at room temperature for 5 h. After completion, H_2_O (500 mL) was added and extracted with EtOAc (200 mLx2 times). The combined organic layers were washed with 20 % aq. HCl solution (200 mL) followed by 8 % aq. NaHCO_3_ solution (200 mL). The organic layer was dried over anhydrous Na_2_SO_4_, filtered, and evaporated under reduced pressure at 45 °C. The crude product was purified using column chromatography (Eluent: EtOAc: hexane, 3 : 7) to afford pure **2** as a white solid (44.2 g, 68.1 %). ^1^H NMR (DMSO‐*d_6_*, 700 MHz): δ 7.98 (s, 1H), 7.92 (s, 1H), 7.81 (s, 2H), 3.64–3.62 (m, 3H), 3.52–3.44 (m, 3H), 3.16–3.15 (m, 2H), 1.40 (s, 9H); ^13^C NMR (DMSO‐*d_6_*, 176 MHz): δ 163.86, 153.77, 140.48, 134.52, 133.33, 132.05, 131.74, 128.09, 79.32, 46.00, 41.13, 27.99; ESMS (*m/z*): 436.2 [M−H]^−^; Elemental analysis for C_16_H_21_Cl_2_N_3_O_5_S Calcd: (%) C 43.84, H 4.83, N 9.59. Found: C 43.79, H 4.80, N 9.53.

#### [(2,4‐dichloro‐5‐sulfamoylphenyl)carbonyl] piperazine trifluoroacetic acid (3)

To a stirred solution of **2** (30.0 g, 77.8 mmol) in DCM (150 mL), TFA (17.9 mL, 233.4 mmol) was added slowly at 0–5 °C. The reaction mixture was allowed to reach room temperature and stirred for an additional 2 h. After completion of the reaction monitored by TLC, the reaction mixture was concentrated under reduced pressure at 40 °C and triturated with diisopropyl ether (100 mLx2 times) to afford **3** as an off‐white solid (22.5 g, 72.3 %). ^1^H NMR (DMSO‐*d_6_*, 700 MHz): δ 9.14 (s, 1H), 9.08 (s, 1H), 8.07 (s, 1H), 8.00 (s, 1H), 7.82 (s, 2H), 3.96–3.92 (m, 1H), 3.76–3.74 (m, 1H), 3.41–3.39 (m, 2H), 3.29–3.06 (m, 4H); ESMS (*m/z*): 338.0 [M+H]^+^ for free base.

#### General procedure for the synthesis of compounds 4–8, 10, 11, 13, 15 and 16

To a stirred solution of **3** (500.0 mg, 1.15 mmol) in DMF (4.0 mL), K_2_CO_3_ (350.0 mg, 2.53 mmol) was added at 15–20 °C. After 15 min stirring, appropriate benzenesulfonyl chloride (1.35 mmol) was added and the reaction mixture was stirred at room temperature (25–35 °C) for 3–4 h. The reaction progress was monitored by TLC. After completion, the reaction mixture was quenched with cold H_2_O (50 mL) and extracted with EtOAc (50 mLx2 times). The combined organic layer was washed with 2 N HCl solution (50 mL) followed by 8 % aq. NaHCO_3_ solution (50 mL), dried over anhydrous Na_2_SO_4_, filtered, and evaporated under reduced pressure at 40 °C. The crude product was recrystallized from the hot diisopropyl ether or a suitable mixture of isopropyl alcohol (IPA) and diisopropyl ether and dried under reduced pressure to afford the title compounds in yields ranging from 77–84 %.

#### General Procedure for the Synthesis of Amino Compounds 9, 12, and 14

To a stirred solution of nitro derivative **8**, or **11**, or **13** (1.0 eq.) in ethanol/H_2_O(1 : 1, 15 mL), iron powder (6 eq.), and aq. HCl (4 eq.) was added and the reaction mixture was heated to 75 °C for 3 h. After completion of the reaction, the reaction mixture was cooled to rt and quenched by saturated aq. NaHCO_3_ solution (60 mL), filtered through a celite cake and concentrated under reduced pressure at 40 °C. The obtained residue was extracted with EtOAc (10 mLx3 times). The combined organic layers were dried over anhydrous Na_2_SO_4_, filtered, and evaporated under reduced pressure at 40 °C. The crude residue was purified by flash chromatography (EtOAc/hexane; 6 : 4) to afford the target compounds in yields ranging from 70 to 75 %.

#### 2,4‐dichloro‐5‐{[4‐(phenylsulfonyl)piperazin‐1‐yl]carbonyl}benzenesulfonamide (4)

Yellow solid (77 %); ^1^H NMR (DMSO‐*d_6_*, 700 MHz): δ 7.88 (s, 1H), 7.86 (s, 1H), 7.76–7.73 (m, 3H), 7.68–7.66 (m, 2H), 7.28 (bs, 2H), 3.77–3.75 (m, 1H), 3.70–3.67 (m, 1H), 3.26–3.24 (m, 2H), 3.08–3.06 (m, 1H), 2.99–2.87 (m, 3H); ^13^C NMR (DMSO‐*d_6_*, 176 MHz): δ 162.77, 142.91, 139.51, 133.13, 132.14, 131.06, 130.92, 130.80, 128.97, 127.28, 126.53, 44.73, 44.50, 44.34, 40.50; ESMS (*m/z*): 478.0 [M+H]^+^; Elemental analysis Calcd for C_17_H_17_Cl_2_N_3_O_5_S_2_: (%) C 42.68, H 3.58, N 8.78. Found: C 42.71, H 3.60, N 8.76.

#### 2,4‐dichloro‐5‐({4‐[(4‐methylphenyl)sulfonyl]piperazin‐1‐yl}carbonyl)benzenesulfonamide (5)

White solid (80 %); ^1^H NMR (DMSO‐*d_6_*, 700 MHz): δ 7.94 (s, 1H), 7.91 (s, 1H), 7.77 (s, 2H), 7.62 (d, *J*=8.2 Hz, 2H), 7.47 (d, *J*=8.1 Hz, 2H), 3.79–3.77 (m, 1H), 3.66–3.63 (m, 1H), 3.26–3.24 (m, 2H), 3.02–2.99 (m, 2H), 2.89–2.87 (m, 2H), 2.41 (s, 3H); ^13^C NMR (DMSO‐*d_6_*, 176 MHz): δ 162.77, 142.91, 139.51, 133.13, 132.14, 131.06, 130.92, 130.80, 128.97, 127.28, 126.53, 44.73, 44.50, 44.34, 40.50; ESMS (*m/z*): 492.0 [M+H]^+^; Elemental analysis Calcd for C_18_H_19_Cl_2_N_3_O_5_S_2_: (%) C 43.91, H 3.89, N 8.53. Found: C 43.89, H 3.88, N 8.51.

#### 2,4‐dichloro‐5‐({4‐[(4‐methoxyphenyl)sulfonyl]piperazin‐1‐yl}carbonyl)benzenesulfonamide (6)

White solid (82 %); ^1^H NMR (DMSO‐*d_6_*, 700 MHz): δ 7.94 (s, 1H), 7.91 (s, 1H), 7.78 (s, 2H), 7.67 (d, *J*=8.8 Hz, 2H), 7.17 (d, *J*=8.8 Hz, 2H), 3.86 (s, 3H), 3.78–3.77 (m, 1H), 3.68–3.67 (m, 1H), 3.26–3.25 (m, 2H), 3.07–2.98 (m, 2H), 2.97–2.87 (m, 2H); ^13^C NMR (DMSO‐*d_6_*, 176 MHz): δ 163.76, 162.91, 140.45, 133.20, 131.96, 131.81, 128.27, 126.37, 114.67, 55.75, 45.76, 45.49, 45.36, 40.51; ESMS (*m/z*): 508.0 [M+H]^+^; Elemental analysis Calcd for C_18_H_19_Cl_2_N_3_O_6_S_2_: (%) C 42.52, H 3.77, N 8.27. Found: C 42.49, H 3.75, N 8.25.

#### 2,4‐dichloro‐5‐({4‐[(4‐chlorophenyl)sulfonyl]piperazin‐1‐yl}carbonyl)benzenesulfonamide (7)

White solid (79 %); ^1^H NMR (DMSO‐*d_6_*, 700 MHz): δ 7.95 (s, 1H), 7.94 (s, 1H), 7.78 (s, 2H), 7.68 (d, *J*=8.8 Hz, 2H), 7.17 (d, *J*=8.7 Hz, 2H), 3.84–3.82 (m, 1H), 3.64–3.61 (m, 1H), 3.27–3.25 (m, 2H), 3.08–3.03 (m, 2H); 2.96–2.90 (m, 2H); ^13^C NMR (DMSO‐*d_6_*, 176 MHz): δ 163.76, 140.44, 138.46, 134.16, 133.97, 133.22, 131.94, 131.83, 129.72, 129.43, 128.37, 45.63, 45.50, 45.25, 40.51; ESMS (*m/z*): 512.0 [M+H]^+^; Elemental analysis Calcd for C_17_H_16_Cl_3_N_3_O_5_S_2_: (%) C 39.82, H 3.14, N 8.19. Found: C 39.79, H 3.16, N 8.17.

#### 2,4‐dichloro‐5‐({4‐[(4‐nitrophenyl)sulfonyl]piperazin‐1‐yl}carbonyl)benzenesulfonamide (8)

Off‐white solid (84 %); ^1^H NMR (700 MHz, DMSO‐*d_6_*): δ 8.46–8.45 (d, *J*=8.3 Hz, 2H), 8.02–8.01 (d, *J*=8.3 Hz, 2H), 7.95 (s, 1H), 7.91 (s, 1H), 7.78 (s, 2H), 3.85–3.83 (m, 1H), 3.64–3.63 (m, 1H), 3.28–3.26 (m, 2H), 3.14–3.12 (m, 2H), 3.04–2.97 (m, 2H); ^13^C NMR (DMSO‐*d_6_*, 176 MHz): δ 163.77, 150.20, 140.86, 140.43, 134.11, 133.25, 131.95, 131.86, 129.10, 128.34, 124.84, 45.62, 45.53, 45.23, 40.52; ESMS: *m/z* 521.0 [M−H]^−^; Elemental analysis Calcd for C_17_H_16_Cl_2_N_4_O_7_S_2_: (%) C 39.01, H 3.08, N 10.71. Found: C 39.03, H 3.06, N 10.69.

#### 5‐({4‐[(4‐aminophenyl)sulfonyl]piperazin‐1‐yl}carbonyl)‐2,4‐dichlorobenzenesulfonamide (9)

Off‐white solid (75 %); ^1^H NMR (DMSO‐*d_6_*, 700 MHz): δ 7.94 (s, 1H), 7.91 (s, 1H), 7.78 (s, 2H), 7.34 (t, *J*=8.6 Hz, 2H), 6.64 (d, *J*=8.7 Hz, 2H), 6.12 (bs, 2H), 3.73–3.68 (m, 2H), 3.25–3.23 (m, 2H), 2.98–2.96 (m, 1H), 2.89–2.76 (m, 3H); ^13^C NMR (DMSO‐*d_6_*, 176 MHz): δ 163.75, 153.40, 140.47, 134.21, 133.19, 131.98, 131.79, 129.56, 128.20, 119.09, 112.77, 56, 45.84, 45.51, 45.41, 40.52; ESMS (*m/z*): 493.0 [M+H]^+^; Elemental analysis Calcd for C_17_H_18_Cl_2_N_4_O_5_S_2_: (%) C 41.38, H 3.68, N 11.36. Found: C 41.40, H 3.70, N 11.37.

#### 5‐({4‐[(4‐acetylaminophenyl)sulfonyl]piperazin‐1‐yl}carbonyl)‐2,4‐dichlorobenzenesulfonamide (10)

Off‐white solid (78 %); ^1^H NMR (DMSO‐*d_6_*, 700 MHz): δ 10.47 (bs, 1H), 7.94 (s, 1H), 7.92 (s, 1H), 7.84–7.83 (m, 2H), 7.78 (s, 2H), 7.67–7.66 (m, 2H), 3.78–3.77 (m, 1H), 3.67–3.61 (m, 1H), 3.26–3.24 (m, 2H), 3.03–2.87 (m, 4H), 2.10 (s, 3H); ^13^C NMR (DMSO‐*d_6_*, 176 MHz): δ 169.15, 163.74, 143.68, 140.45, 134.16, 133.18, 131.94, 131.79, 128.76, 128.27, 128.25, 118.71, 45.75, 45.49, 45.36, 40.50, 24.15; ESMS (*m/z*): 535.0 [M+H]^+^; Elemental analysis Calcd for C_19_H_20_Cl_2_N_4_O_6_S_2_: (%) C 42.62, H 3.77, N 10.46. Found: C 42.58, H 3.75, N 10.43.

#### 2,4‐dichloro‐5‐({4‐[(3‐nitrophenyl)sulfonyl]piperazin‐1‐yl}carbonyl)benzenesulfonamide (11)

Off‐white solid (81 %); ^1^H NMR (DMSO‐*d_6_*, 700 MHz): δ 8.58–8.57 (m, 1H), 8.38 (s, 1H), 8.19–8.18 (m, 1H), 7.98–7.96 (m, 2H), 7.90 (s, 1H), 7.77 (s, 2H), 3.85–3.84 (m, 1H), 3.64–3.62 (m, 1H), 3.28–3.27 (m, 2H), 3.18–3.17 (m, 1H), 3.14–3.12 (m, 1H), 3.05–3.03 (m, 1H), 2.98–2.89 (m, 1H); ^13^C NMR (DMSO‐*d_6_*, 176 MHz): δ 163.78, 148.20, 140.43, 136.86, 134.09, 133.43, 133.26, 131.96, 131.87, 131.70, 128.33, 128.05, 122.14, 45.65, 45.49, 45.26, 40.50; ESMS: *m/z* 523.0 [M+H]^+^; Elemental analysis Calcd for C_17_H_16_Cl_2_N_4_O_7_S_2_: (%) C 39.01, H 3.08, N 10.71. Found: C 38.97, H 3.04, N 10.76.

#### 5‐({4‐[(3‐aminophenyl)sulfonyl]piperazin‐1‐yl}carbonyl)‐2,4‐dichlorobenzenesulfonamide (12)

Off‐white solid (73 %); ^1^H NMR (DMSO‐*d_6_*, 700 MHz): δ 7.94 (s, 1H), 7.92 (s, 1H), 7.78 (s, 2H), 7.25 (t, *J*=8.6 Hz, 1H), 6.89 (s, 1H), 6.84–6.83 (d, *J*=8.6 Hz, 1H), 6.78–6.77 (d, *J*=8.6 Hz, 1H), 5.66 (bs, 2H), 3.75–3.69 (m, 2H), 3.26–3.25 (m, 2H), 3.05–2.95 (m, 2H), 2.91–2.83 (m, 2H); ^13^C NMR (DMSO‐*d_6_*, 176 MHz): δ 163.79, 149.65, 140.47, 135.28, 134.20, 133.18, 131.96, 131.80, 129.80, 128.24, 118.10, 113.94, 11.56, 45.82, 45.54, 45.44, 40.58; ESMS: *m/z* 493.0 [M+H]^+^; Elemental analysis Calcd for C_17_H_18_Cl_2_N_4_O_7_S_2_: (%) C 41.38, H 3.68, N 11.36. Found: C 41.35, H 3.65, N 11.38.

#### 2,4‐dichloro‐5‐({4‐[(2‐nitrophenyl)sulfonyl]piperazin‐1‐yl}carbonyl)benzenesulfonamide (13)

White solid (77 %); ^1^H NMR (DMSO‐*d_6_*, 700 MHz): δ 8.02–8.00 (m, 2H), 7.98–7.93 (m, 3H), 7.89–7.87 (m, 1H), 7.78 (s, 2H), 3.84–3.82 (m, 1H), 3.66–3.64 (m, 1H), 3.29–3.28 (m, 2H), 3.21–3.20 (m, 2H), 3.05–3.03 (m, 1H), 2.97–2.98 (m, 1H); ^13^C NMR (DMSO‐*d_6_*, 176 MHz): δ 163.84, 147.86, 140.45, 135.06, 134.18, 133.27, 132.49, 131.96, 130.43, 129.04, 128.41, 124.33, 45.82, 45.52, 45.16, 40.85; ESMS (*m/z*): 523.0 [M+H]^+^; Elemental analysis Calcd. for C_17_H_16_Cl_2_N_4_O_7_S_2_: (%) C 39.01, H 3.08, N 10.71. Found: C 39.04, H 3.06, N 10.68.

#### 5‐({4‐[(2‐aminophenyl)sulfonyl]piperazin‐1‐yl}carbonyl)‐2,4‐dichlorobenzenesulfonamide (14)

Off‐white solid (70 %); ^1^H NMR (DMSO‐*d_6_*, 700 MHz): δ 7.94 (s, 1H), 7.93 (s, 1H), 7.78 (s, 2H), 7.39–7.37 (m, 1H), 7.34–7.31 (m, 1H), 6.87–6.86 (m, 1H), 6.65 (t, 1H), 6.06 (bs, 2H), 3.71–3.65 (m, 2H), 3.28–3.26 (m, 2H), 3.18–3.16 (m, 1H), 3.07–3.01 (m, 2H), 2.98–2.95 (m, 1H); ^13^C NMR (DMSO‐*d_6_*, 176 MHz): δ 163.77, 147.44, 140.46, 134.44, 134.20, 133.20, 131.98, 131.79, 129.77, 128.24, 117.44, 115.38, 114.91, 45.55, 45.51, 45.16, 40.55; ESMS (*m/z*): 493.1 [M+H]^+^, Elemental analysis Calcd for C_17_H_18_Cl_2_N_4_O_7_S_2_: (%) C 41.38, H 3.68, N 11.36. Found: C 41.32, H 3.65, N 11.32.

#### 2,4‐dichloro‐5‐({4‐[(3,4‐dichlorophenyl)sulfonyl]piperazin‐1‐yl}carbonyl)benzenesulfonamide (15)

White solid (75 %); ^1^H NMR (DMSO‐*d_6_*, 700 MHz): δ 8.05 (s, 3H), 7.95–7.94 (m, 1H), 7.78 (s, 2H), 7.72–7.70 (m, 2H), 3.90–3.89 (m, 1H), 3.58–3.56 (m, 1H), 3.27–3.25 (m, 2H), 3.20–3.18 (m, 1H), 3.06–3.04 (m, 2H), 2.94–2.92 (m, 1H); ^13^C NMR (DMSO‐*d_6_*, 176 MHz): δ 163.74, 140.42, 136.66, 135.61, 134.15, 133.25, 132.61, 131.92, 131.83, 129.13, 128.44, 127.60, 45.56, 45.47, 45.19, 40.47; ESMS (*m/z*): 565.0 [M+H_3_O]^+^; Elemental analysis Calcd for C_17_H_15_Cl_4_N_3_O_5_S_2_: (%) C 37.31, H 2.76, N 7.68. Found: C 37.29, H 2.78, N 7.66.

#### 5‐{[4‐(benzylsulfonyl)piperazin‐1‐yl]carbonyl}‐2,4‐dichlorobenzenesulfonamide (16)

Off‐white solid (81 %); ^1^H NMR (DMSO‐*d_6_*, 700 MHz): δ 7.99 (s, 1H), 7.93 (s, 1H), 7.82 (s, 2H), 7.42–7.36 (m, 5H), 4.47 (s, 2H), 3.76–3.74 (m, 1H), 3.59–3.58 (m, 1H), 3.18–3.13 (m, 2H), 3.01–2.89 (m, 4H); ^13^C NMR (DMSO‐*d_6_*, 176 MHz): δ 163.83, 140.50, 134.30, 133.27, 132.06, 131.86, 130.91, 129.21, 128.43, 128.32, 128.14, 54.75, 46.24, 45.57, 45.06, 41.20; ESMS (*m/z*): 492.0 [M+H]^+^; Elemental analysis Calcd for C_18_H_19_Cl_2_N_3_O_5_S_2_: (%) C 43.91, H 3.89, N 8.53. Found: C 43.88, H 3.87, N 8.54.

### *In Vitro* Carbonic Anhydrase Inhibition Assay

An Applied Photophysics stopped‐flow instrument was used to evaluate the ability of the test compounds to inhibit the CA‐catalyzed CO_2_ hydration.^[20]^ Phenol red (at a concentration of 0.2 mM) was used as an indicator, working at the absorbance maximum of 557 nm, with 20 mM HEPES (pH 7.4) as a buffer, and 20 mM Na_2_SO_4_ (to maintain constant ionic strength), following the initial rates of the CA‐catalyzed CO_2_ hydration reaction for a period of 10–100 s. The CO_2_ concentrations ranged from 1.7 to 17 mM for the assessment of the kinetic parameters and inhibition constants. Enzyme concentrations varied between 5 and 12 nM.[^25^, ^26^] For each inhibitor, at least six traces of the initial 5–10 % of the reaction were used to determine the initial velocity. The uncatalyzed rates were calculated in the same manner and subtracted from the total observed rates. Stock solutions of each inhibitor (0.1 mM) were prepared in distilled‐deionized H_2_O and dilutions up to 0.01 nM were done thereafter with the assay buffer. Inhibitor and enzyme solutions were preincubated together for 15 min at room temperature prior to the assay, to allow for the formation of the E–I complex.^[27]^ The inhibition constants were obtained by non‐linear least‐squares methods using PRISM 3^[28]^ and the Cheng‐Prusoff equation as reported earlier^[29]^ and represent the mean from at least three different determinations. hCAs I and II were purchased from Merck, while hCAs IX and XII are recombinant and obtained *in‐house*.[^30^, ^31^, ^32^, ^33^]

### Animal Model and Intraocular Pressure Studies

Male New Zealand Albino (NZW) rabbits (body weight 2–2.5 kg) were used in this study. The animals were housed in individual cages with food and water ad libitum, maintained on 12–12 h light/dark cycle in a temperature controlled room (22–23 °C) and numbered consecutively with a tattoo in the ear. Prior to starting the study, all animals were subjected to a general and ophtalmic examination. All experiments were performed in accordance with the European Regulations (Council Directive of the European Community 2010/63/EU), upon authorizations of Italian Ministry of Health (authorization number 110/2021‐PR) and with the Italian regulation on protection of animals used for experimental purpose (Italian Legislative Decree 26, March 13, 2014). A stock solution of compounds was prepared in DMSO, then dissolved in sterile solution (0.9 % NaCl) to a final concentration of 1 %. To obtain the acute ocular hypertension model, 50 μl of a sterile hypertonic saline (5 % NaCl dissolved in sterile water) was injected into the vitreous bilaterally as previously described.^[34]^ The animals were locally anesthetized with one drop of 0.4 % (4 mg/ml) oxybuprocaine hydrochloride and immediately before each set of pressure measurements. The IOP was measured, using a pneumo‐tonometer Reichert model T30 (Reichert, Inc., Depew, NY), in basal condition, after the hypertension stabilization (10 minutes after injection) and at different timepoints after administration of compounds (30, 60, 120 and 240 min). All IOP measurements were done by the same investigators using the same tonometer. The efficacy of the different drugs in lowering IOP was evaluated after drug administration and normalized with vehicles. The treatment was performed in six animals per drug and compared to vehicle (0.9 % NaCl, 0.1 % DMSO) and the reference drug acetazolamide. At the end of the experiments, the animals were pre‐anesthetized with xilazine (Xilor 2 %, 5 mg/kg) plus ketamine (Lobotor 20 mg/kg) i.m. and euthanized with an overdose of anesthetic (Pentothal sodium 0.15 g/kg, i. v. bolus).^[35]^

### Molecular Modelling

The crystallographic structure of hCA II in complex with 2‐(4‐benzhydrylpiperazin‐1‐yl)‐N‐(4‐sulfamoylphenyl)acetamide (PDB‐ID: 6ZR8)^[23]^ was retrieved from the Protein Data Bank (RCBS.org, PDB) and was used as a rigid receptor in molecular docking simulations. Molecule 7 and 9 were sketched in 2D with the Picto software (version 4.5.4.1, OpenEye Cadence Molecular Sciences, Santa Fe, NM)^[36]^ and converted into a 3D structure with OMEGA (version 4.2.0.1, OpenEye Cadence Molecular Sciences, Santa Fe, NM).[^37^, ^38^] The protonation state of the molecules was predicted at pH 7.4 using the pKa prediction software MOKA,[^39^, ^40^, ^41^] whereas the sulfonamide group was manually deprotonated to mimic Zn (II) coordination in agreement with previous studies.[^15^, ^42^] Ligand energy minimization was performed with SZYBKI (version 2.5.0.1, OpenEye Cadence Molecular Sciences, Santa Fe, NM).^[42]^ Molecular docking was carried out with the GOLD program (The Cambridge Crystallographic Data Centre) version 2023.1.012.[^43^, ^44^] The spherical binding site had a radius of 14 Å and it was centered on the catalytic Zn (II) ion; the CHEMSCORE docking function was used.

## Author Contributions

The manuscript was written through the contributions of all authors. All authors approved the final version of the manuscript.

## Conflict of Interests

The authors declare that there are no conflicts of interests.

1

## Supporting information

As a service to our authors and readers, this journal provides supporting information supplied by the authors. Such materials are peer reviewed and may be re‐organized for online delivery, but are not copy‐edited or typeset. Technical support issues arising from supporting information (other than missing files) should be addressed to the authors.

Supporting Information

## Data Availability

The data that support the findings of this study are available in the supplementary material of this article.
