# Supplementary material for: Lasamide Containing Sulfonylpiperazines as Effective Agents for the Management of Glaucoma Associated Symptoms
Source: ChemMedChem. 2024 Nov 8;19(24):e202400601. doi: 10.1002/cmdc.202400601 (PMC11648825; doi:10.1002/cmdc.202400601)

ChemMedChem

Supporting Information

Lasamide Containing Sulfonylpiperazines as Effective Agents for the Management of Glaucoma Associated Symptoms

Jaydeo T. Kilbile, Suryakant B. Sapkal, Gioele Renzi, Ilaria D'Agostino, Mohamed Boudjelal, Yasinalli Tamboli,* Luigi Cutarella, Mattia Mori, Silvia Sgambellone, Serafina Villano, Silvia Marri, Laura Lucarini, Simone Carradori, Fabrizio Carta,* and Claudiu T. Supuran

Lasamide Containing Sulfonylpiperazines as Effective Agents for the Management of Glaucoma Associated Symptoms

Jaydeo T. Kilbile[a], Suryakant B. Sapkal[a], Gioele Renzi[b], Ilaria D'Agostino[b,c,g], Mohamed Boudjelal[d], Yasinalli Tamboli[d]*, Luigi Cutarella[e], Mattia Mori[e], Silvia Sgambellone[f], Serafina Villano[f] , Silvia Marri[f] , Laura Lucarini[f], Simone Carradori[g], Fabrizio Carta[b]* and Claudiu T. Supuran[b]

[a.] Department of Chemistry, School of Basic and Applied Sciences, MGM University, Chhatrapati Sambhajinagar, 431003, MS, India

[b.] NEUROFARBA Department, Sezione di Scienze Farmaceutiche e Nutraceutiche, University of Florence, 50019, Sesto Fiorentino, Florence, Italy

[c.] Department of Pharmacy, University of Pisa, 56126, Pisa, Italy

[d.] King Abdullah International Medical Research Center (KAIMRC), King Saud Bin Abdulaziz University for Health Sciences, Ministry of National Guard-Health Affairs, Riyadh 14811, Saudi Arabia

Corresponding authors: YT, yasinmedchem@gmail.com; FC, fabrizio.carta@unifi.it

Figure S1: Compound **2** (^{1}H NMR, DMSO-d_6, 700 MHz)

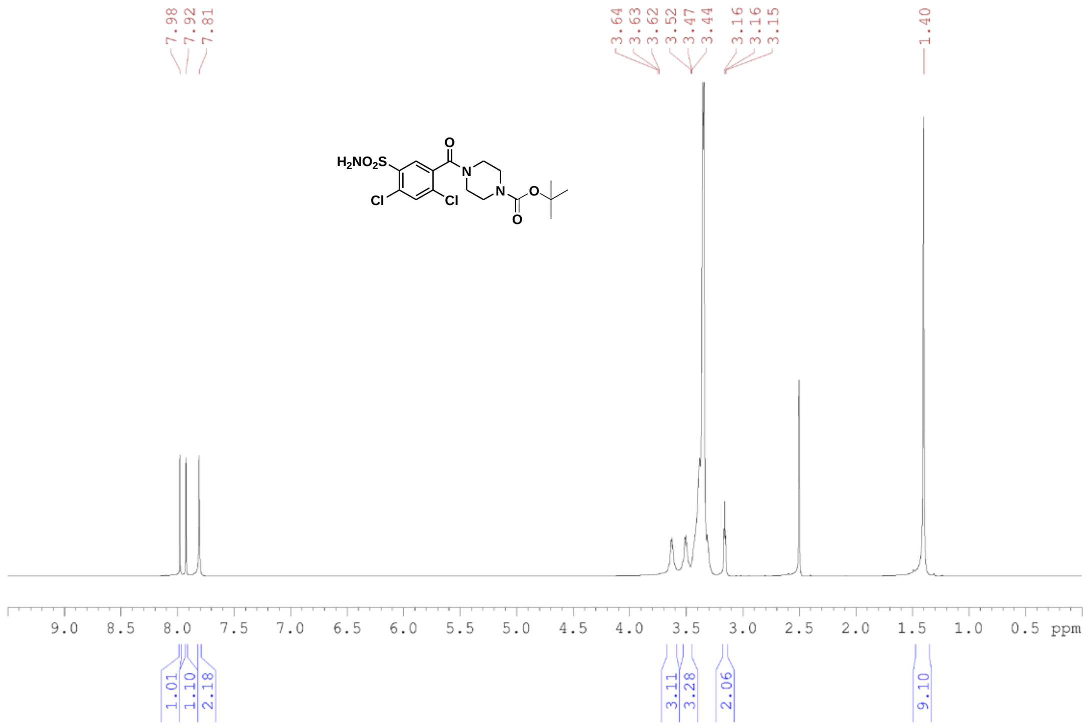

Figure S2: Compound **2** (ESMS)

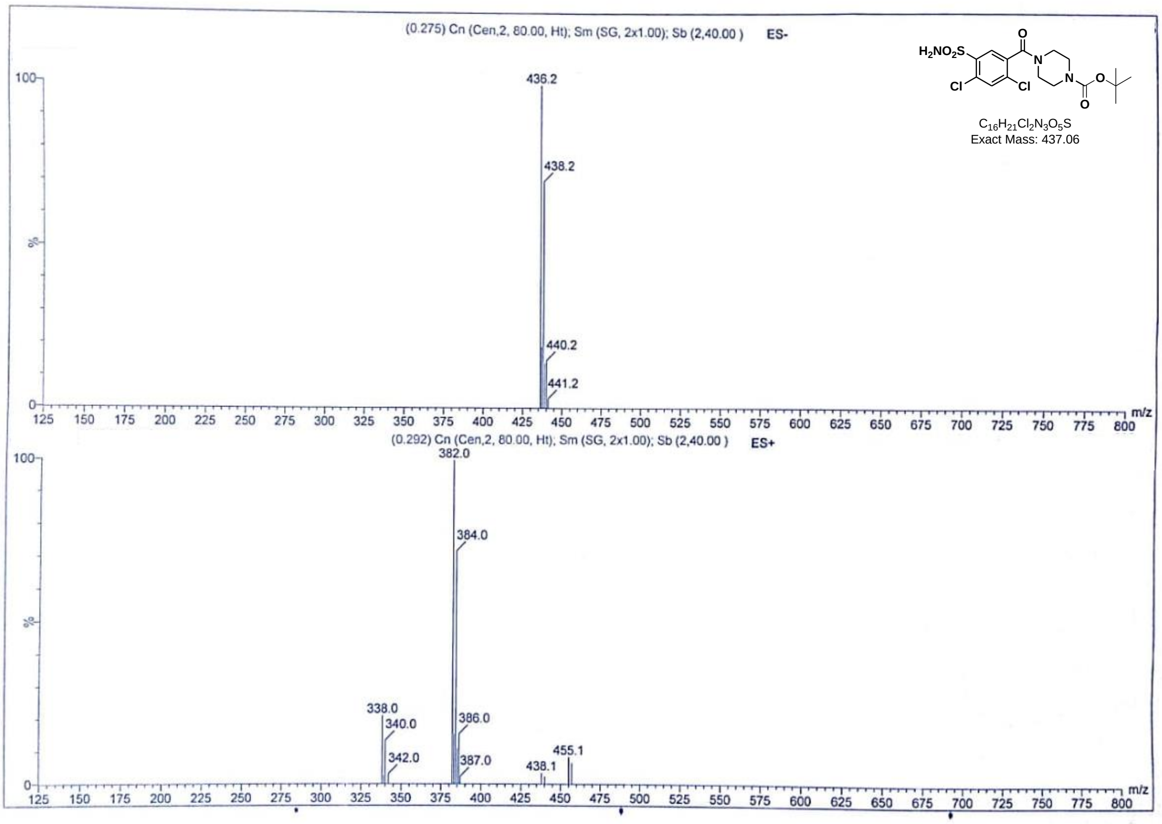

Figure S3: Compound **3** (^{1}H NMR, DMSO-d_6, 700 MHz)

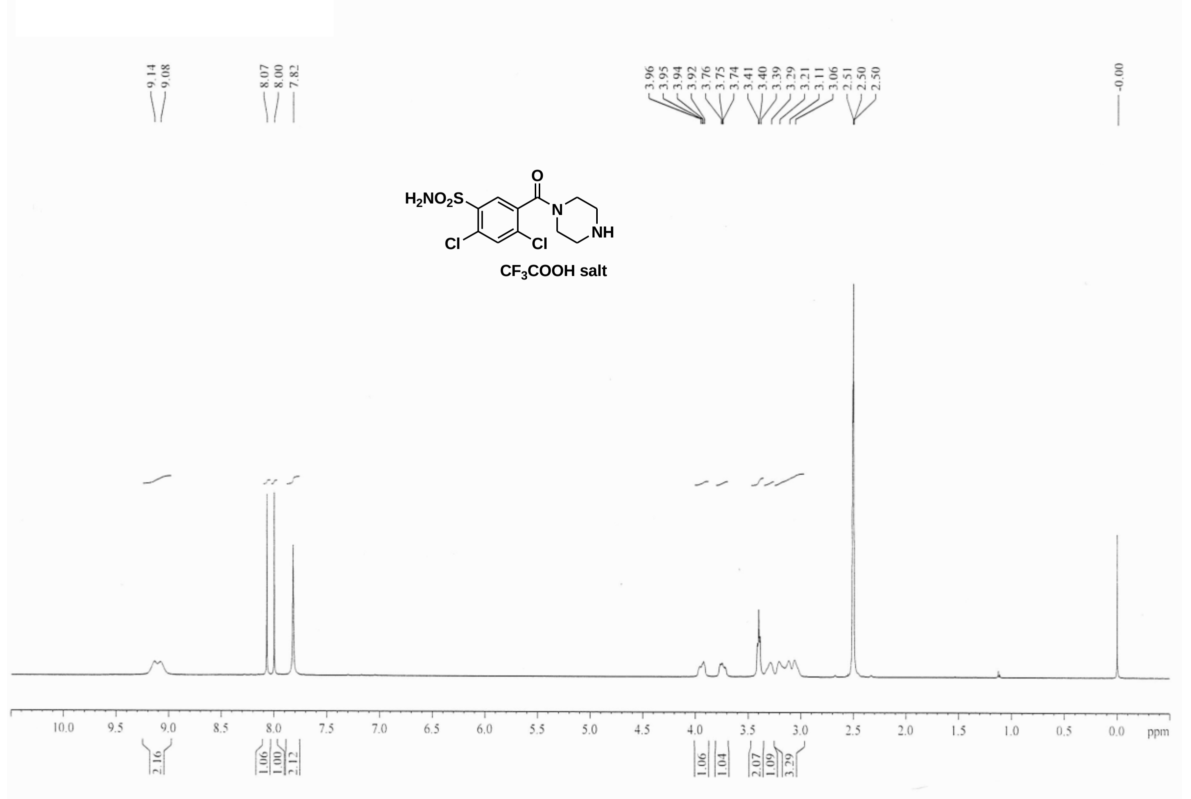

Figure S4: Compound **3** (ESMS)

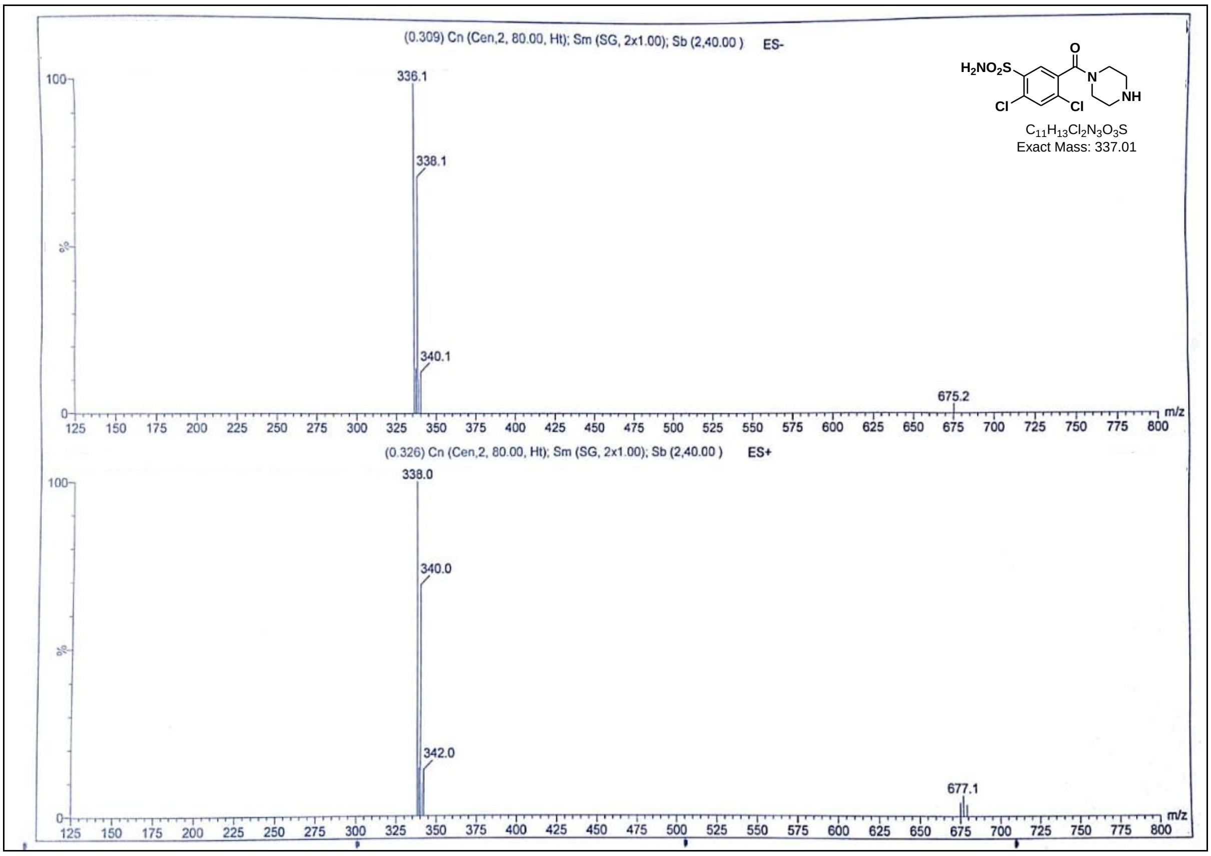

Figure S5: Compound **4** (^{1}H NMR, DMSO-d_6, 700 MHz)

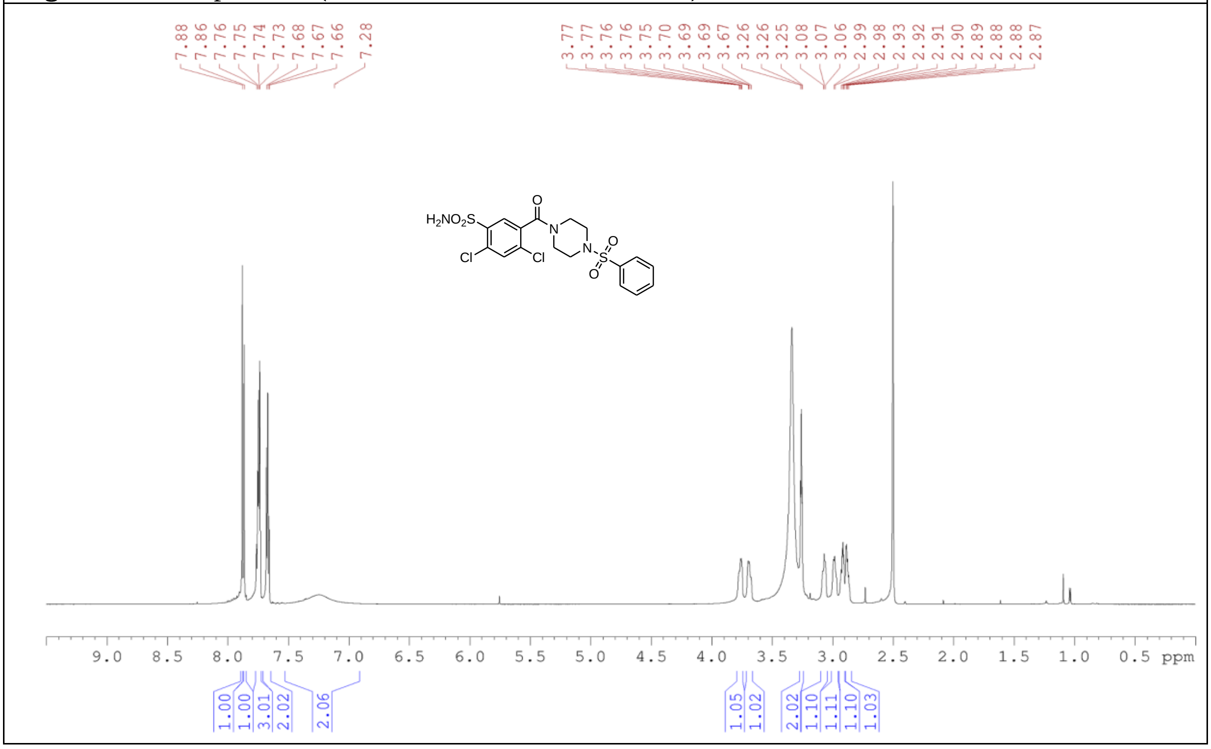

Figure S6: Compound **4** (^{13}C NMR, DMSO-d_6, 176 MHz)

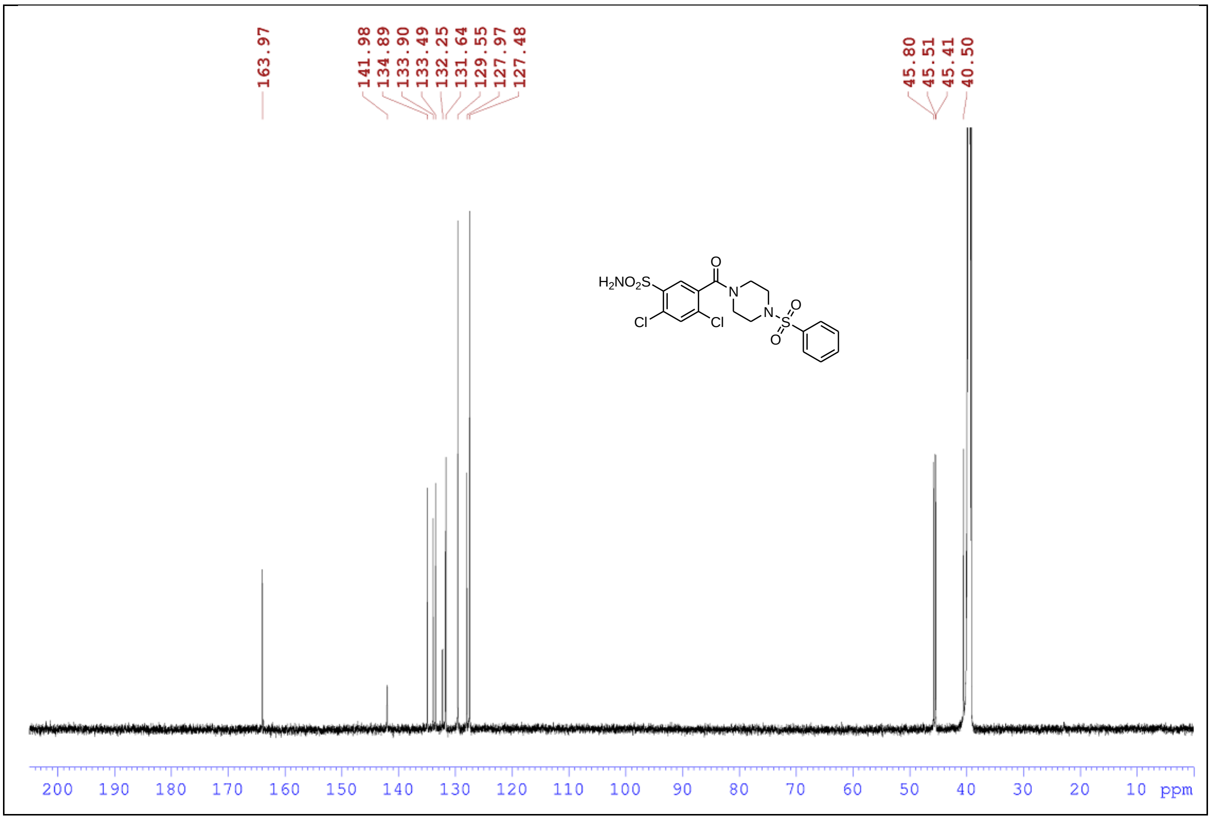

Figure S7: Compound **4** (ESMS)

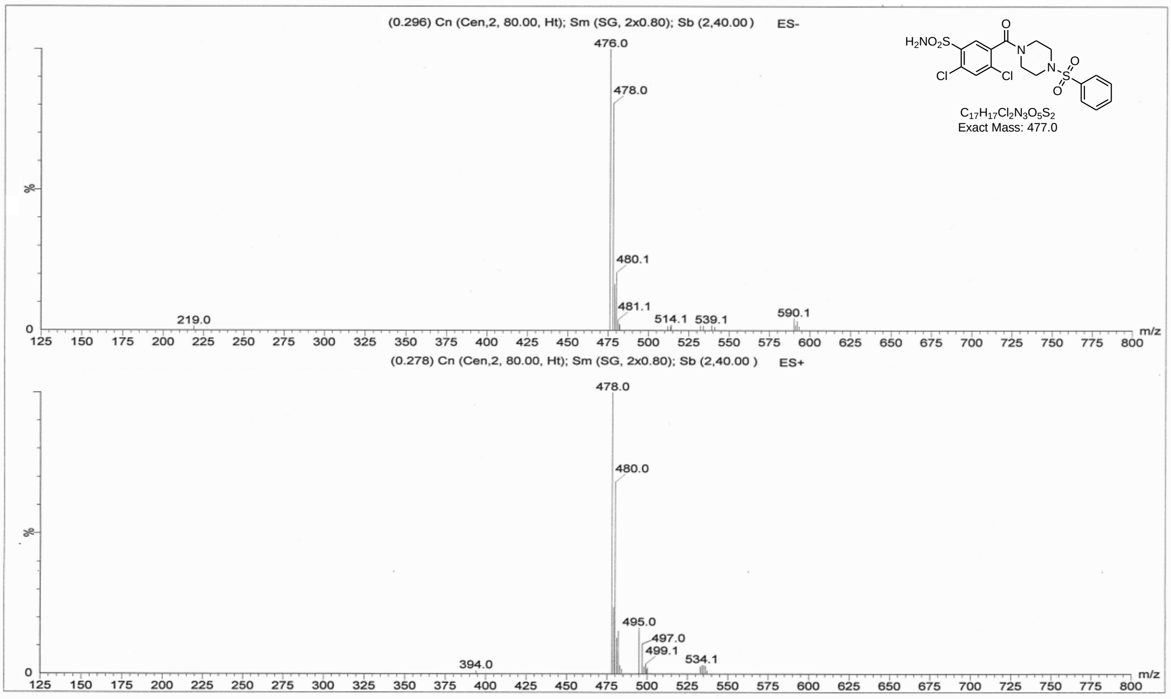

Figure S8: Compound **5** (^{1}H NMR, DMSO-d_6, 700 MHz)

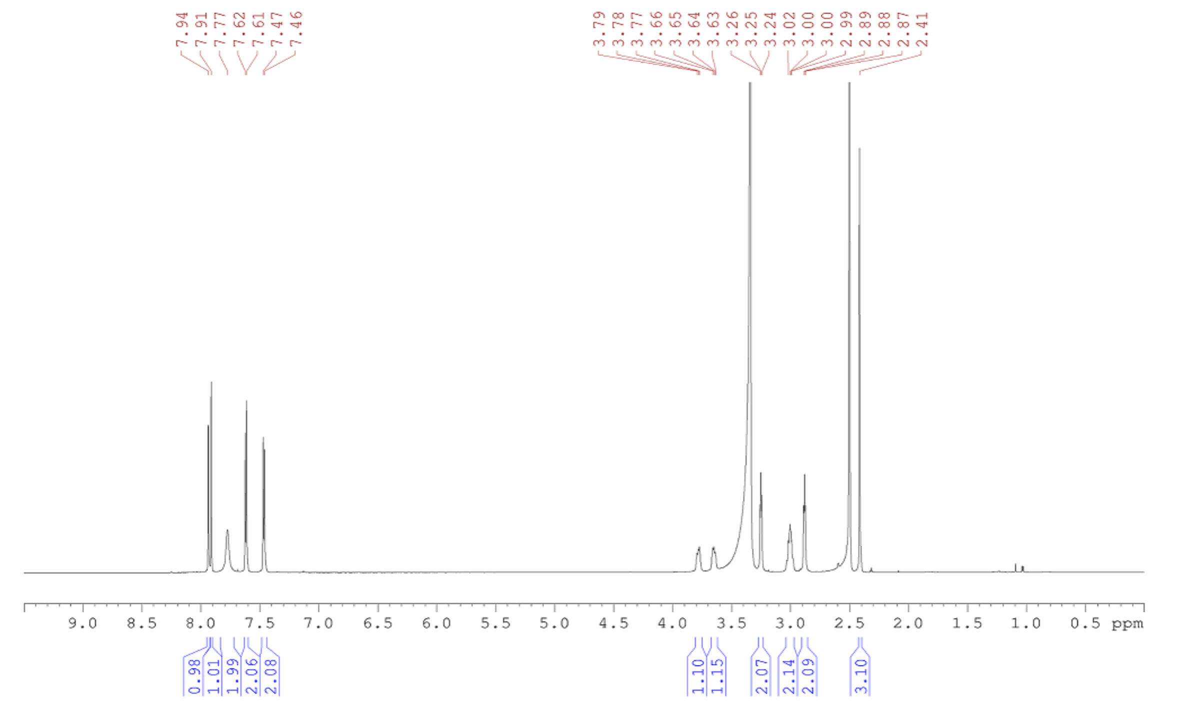

Figure S9: Compound **5** (^{13}C NMR, DMSO-d_6, 176 MHz)

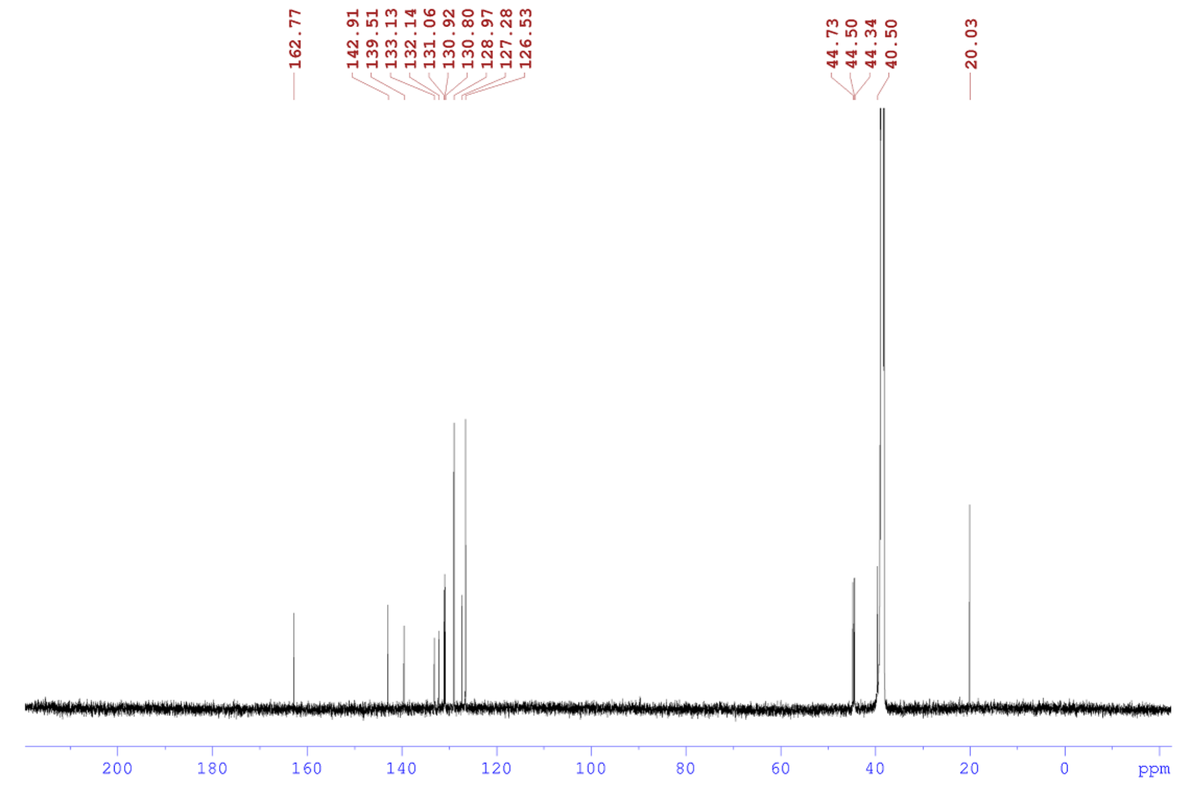

Figure S10: Compound **5** (ESMS)

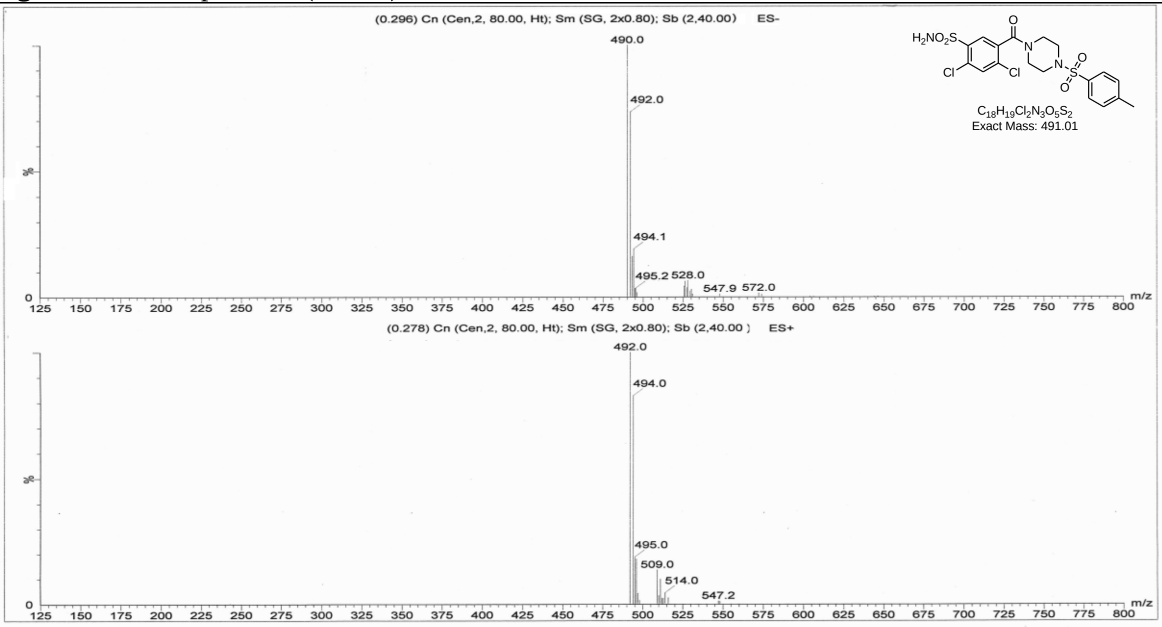

Figure S11: Compound **6** (^{1}H NMR, DMSO-d_6, 700 MHz)

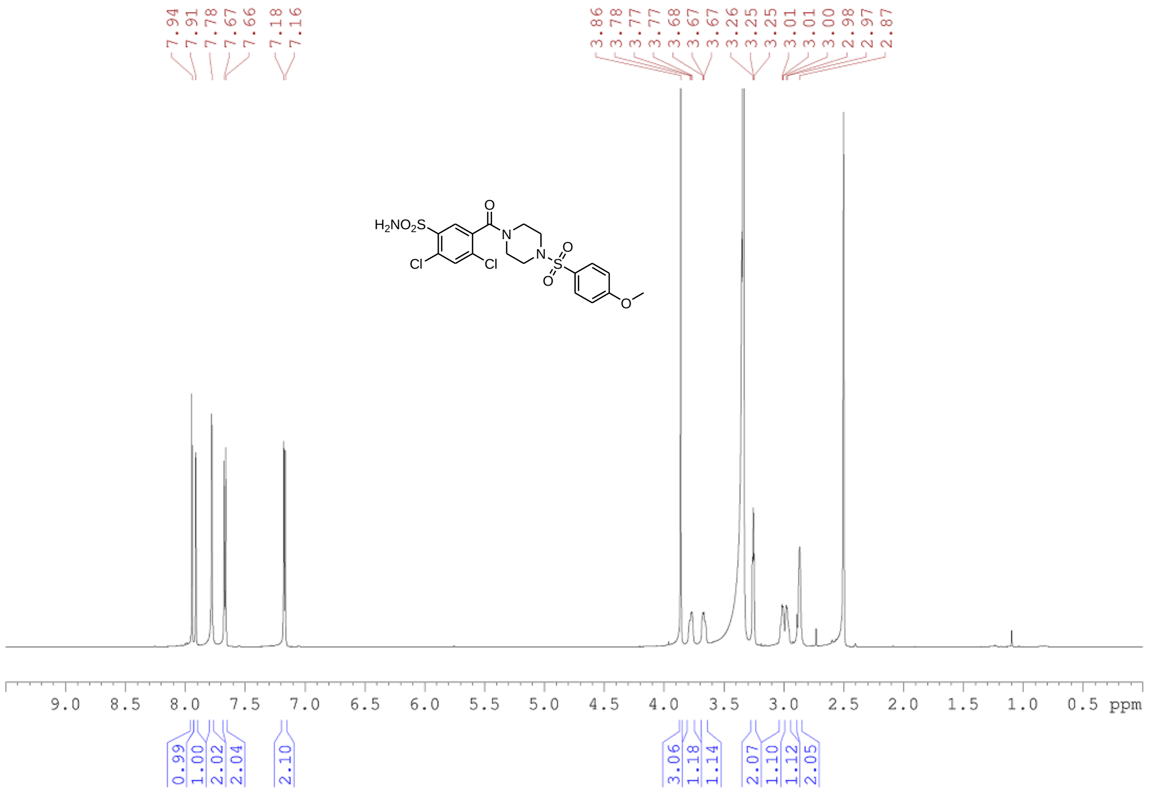

Figure S12: Compound **6** (^{13}C NMR, DMSO-d_6, 176 MHz)

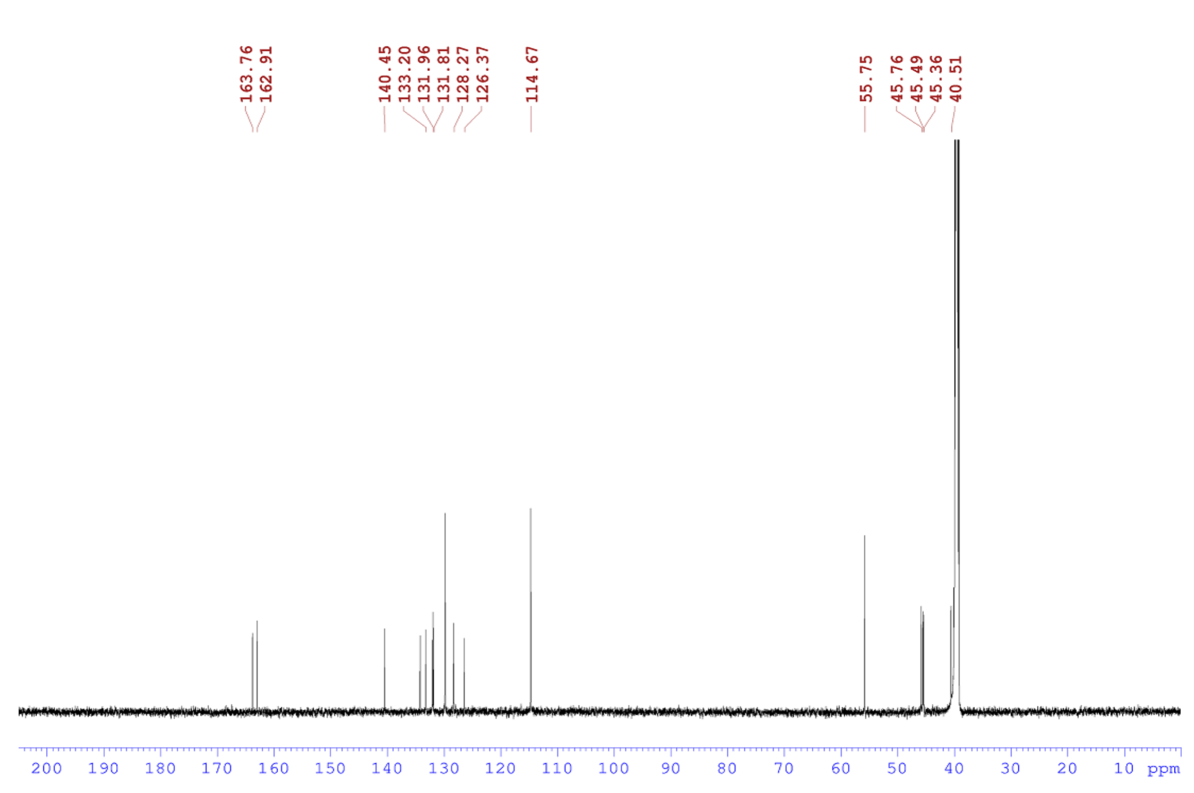

Figure S13: Compound **6** (ESMS)

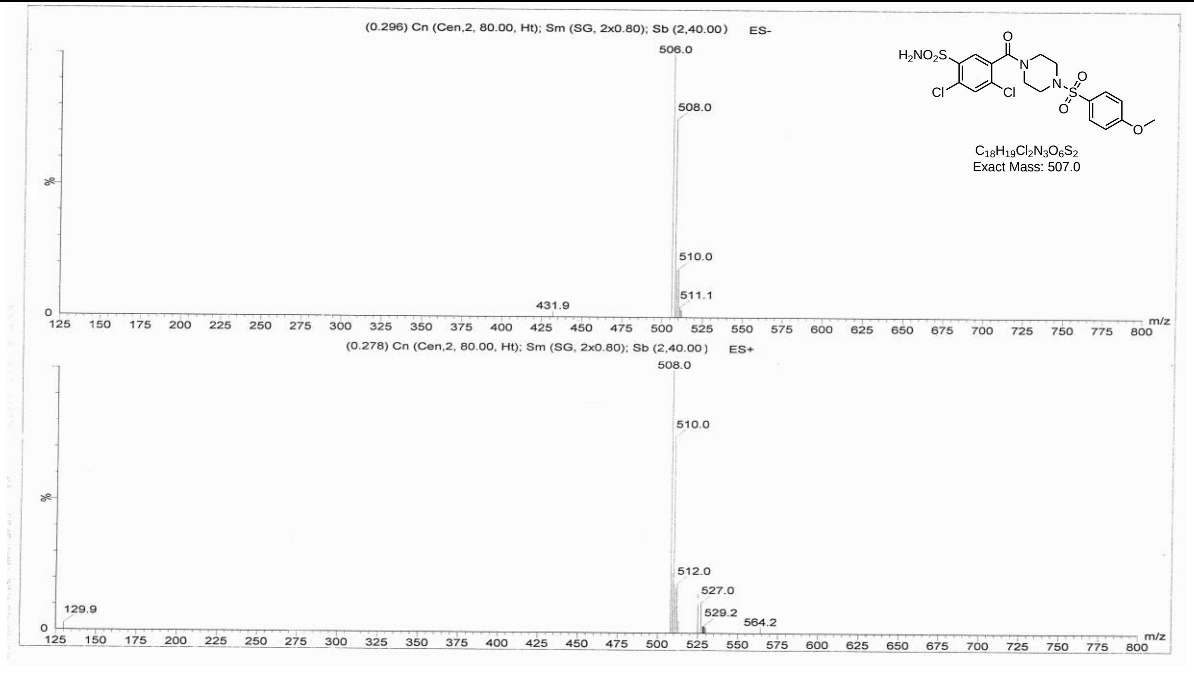

Figure S14: Compound **7** (^{1}H NMR, DMSO-d_6, 700 MHz)

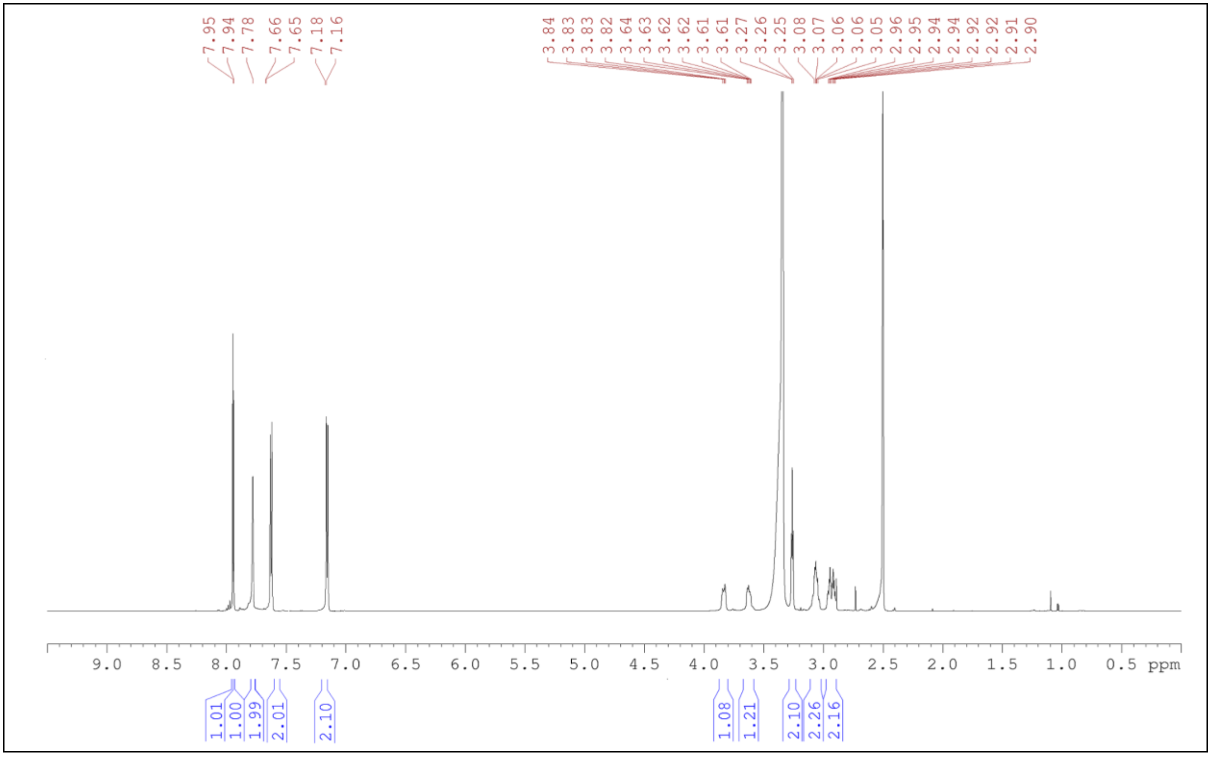

Figure S15: Compound **7** (^{13}C NMR, DMSO-d_6, 176 MHz)

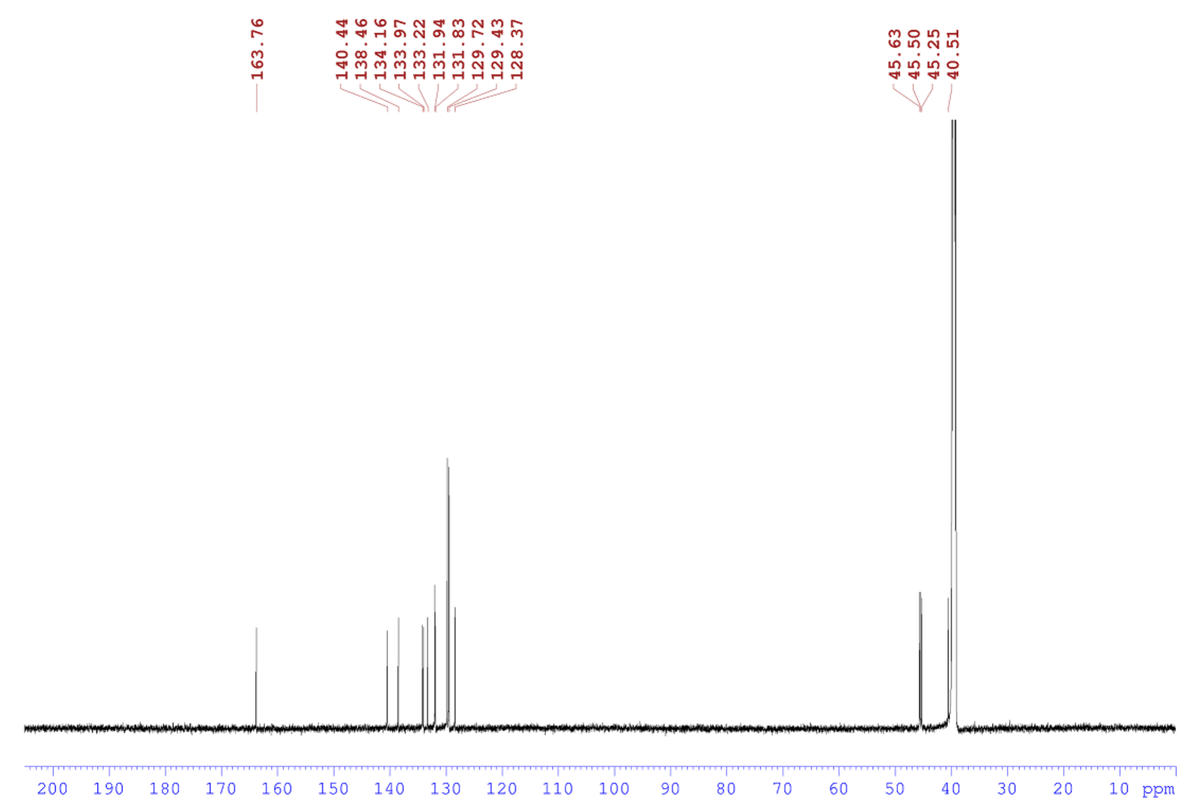

Figure S16: Compound **7** (ESMS)

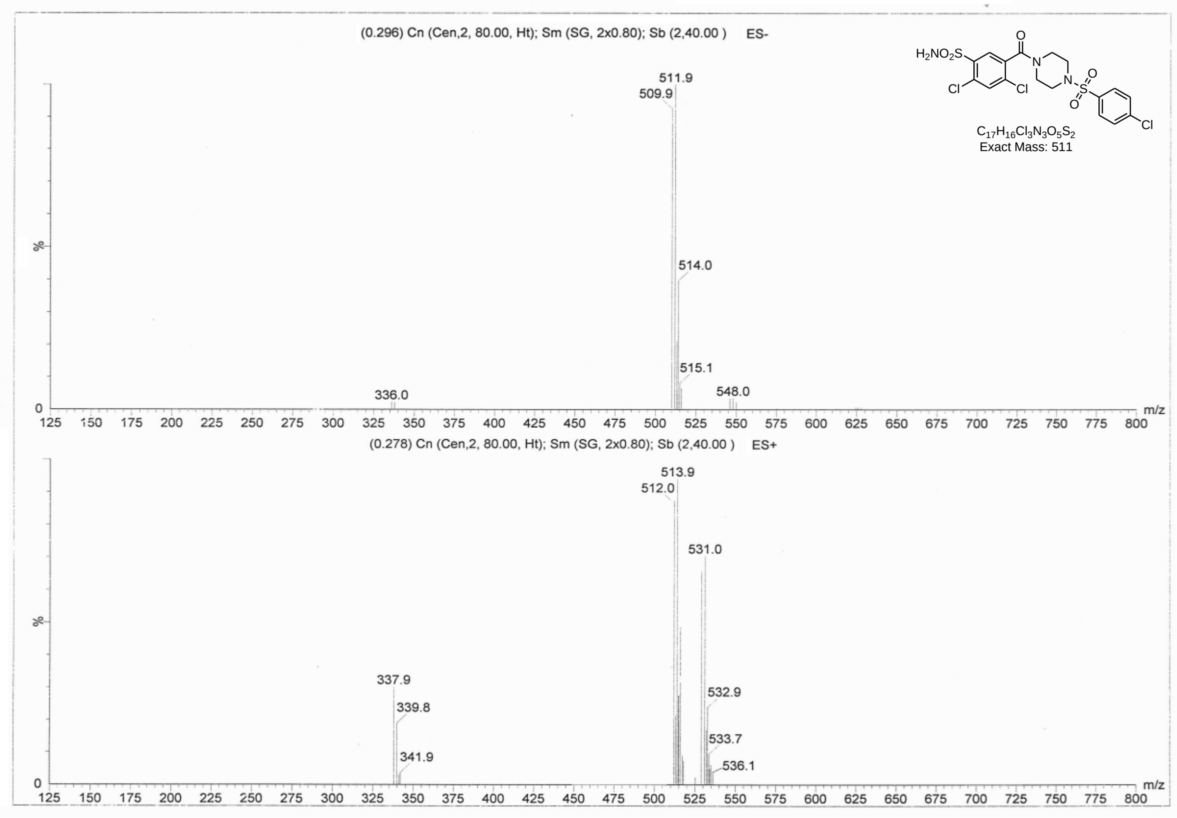

Figure S17: Compound **8** (^{1}H NMR, DMSO-d_6, 700 MHz)

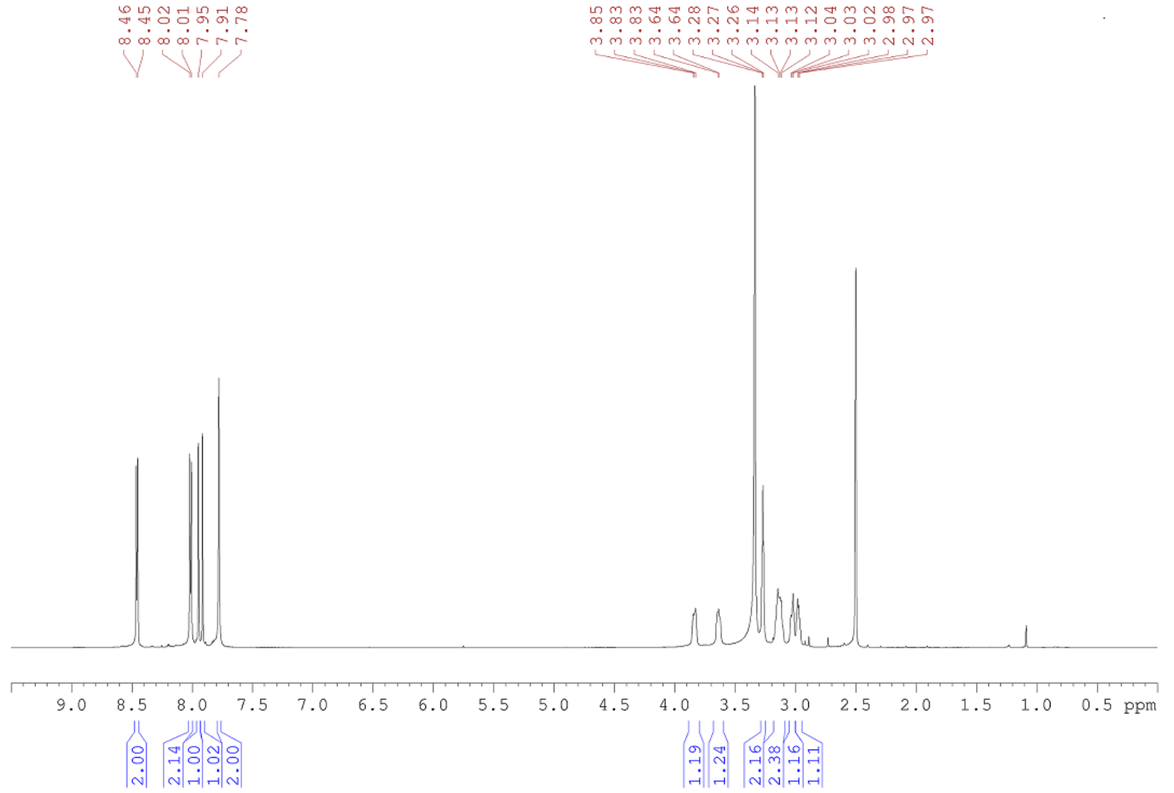

Figure S18: Compound **8** (^{13}C NMR, DMSO-d_6, 176 MHz)

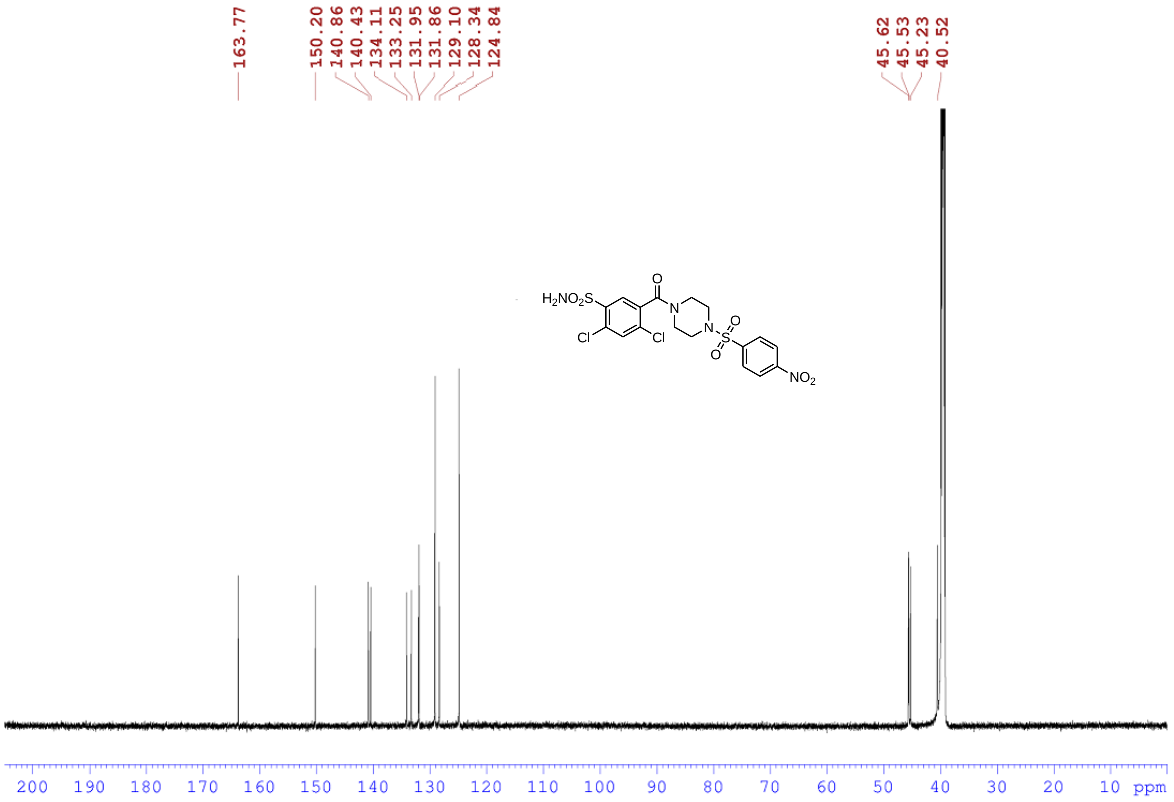

Figure S19: Compound **8** (ESMS)

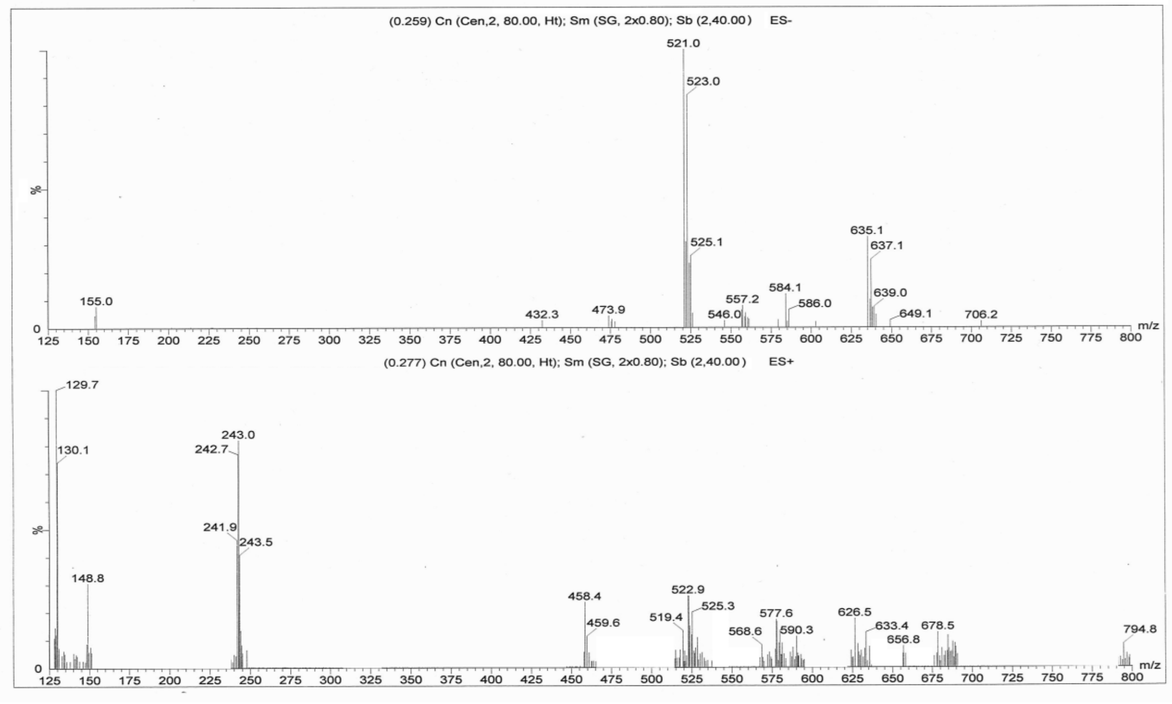

Figure S20: Compound **9** (^{1}H NMR, DMSO-d_6, 700 MHz)

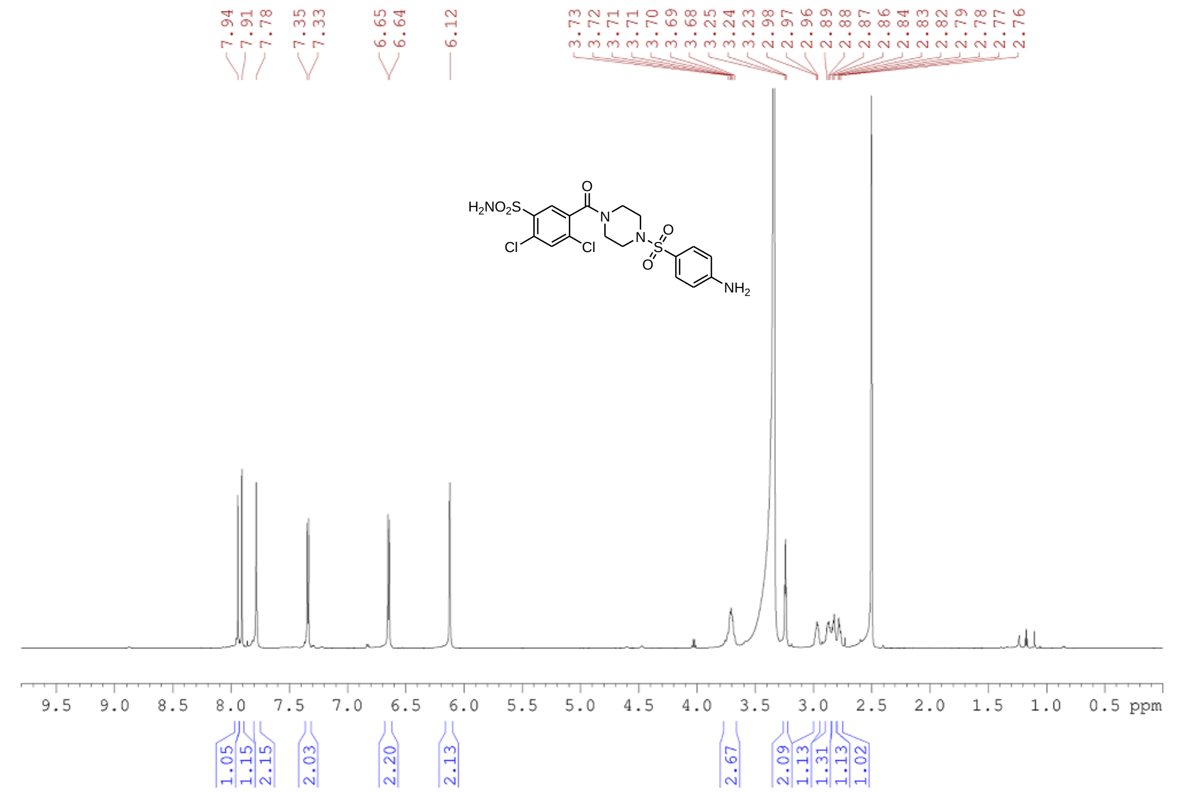

Figure S21: Compound **9** (^{13}C NMR, DMSO-d_6, 176 MHz)

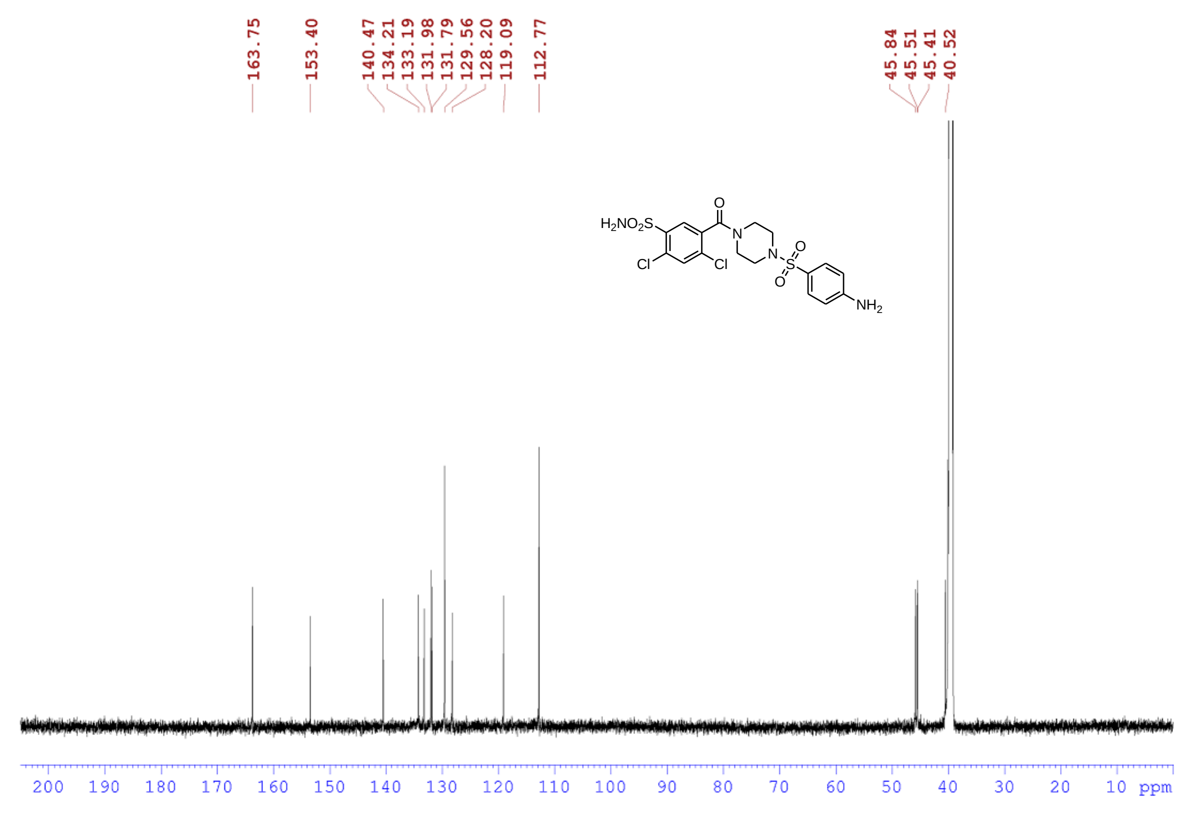

Figure S22: Compound **9** (ESMS)

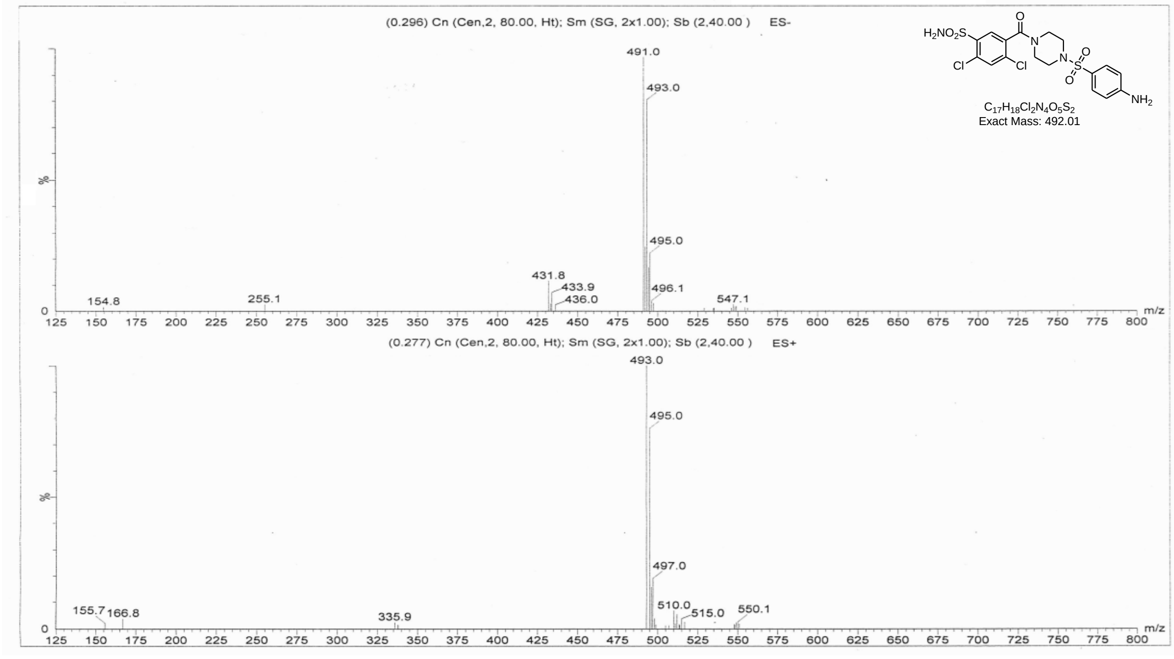

Figure S23: Compound **10** (^{1}H NMR, DMSO-d_6, 700 MHz)

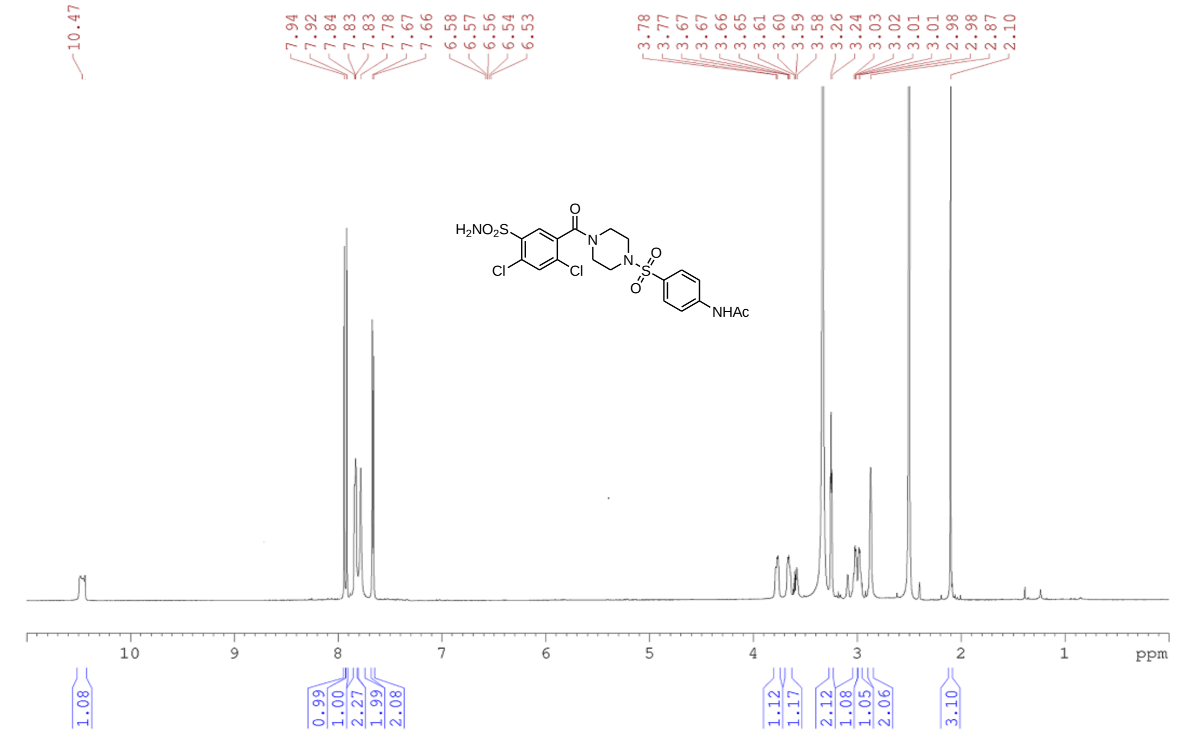

Figure S24: Compound **10** (^{13}C NMR, DMSO-d_6, 176 MHz)

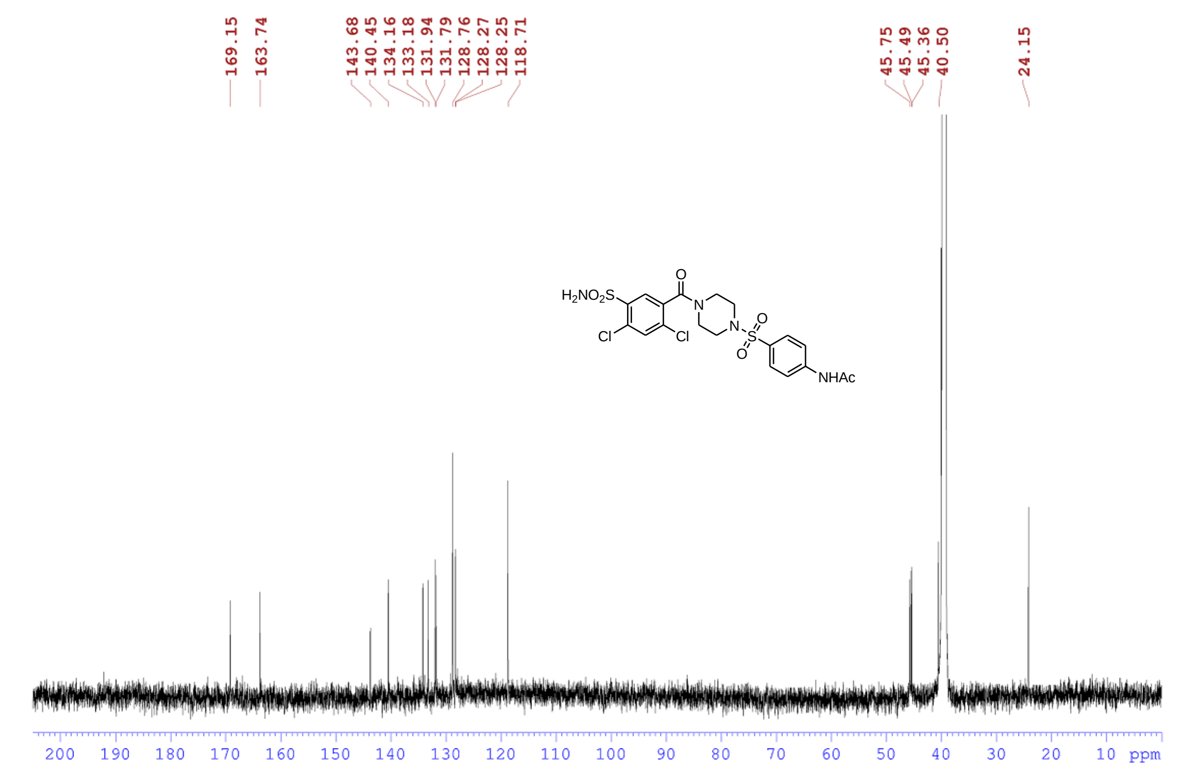

Figure S25: Compound **10** (ESMS)

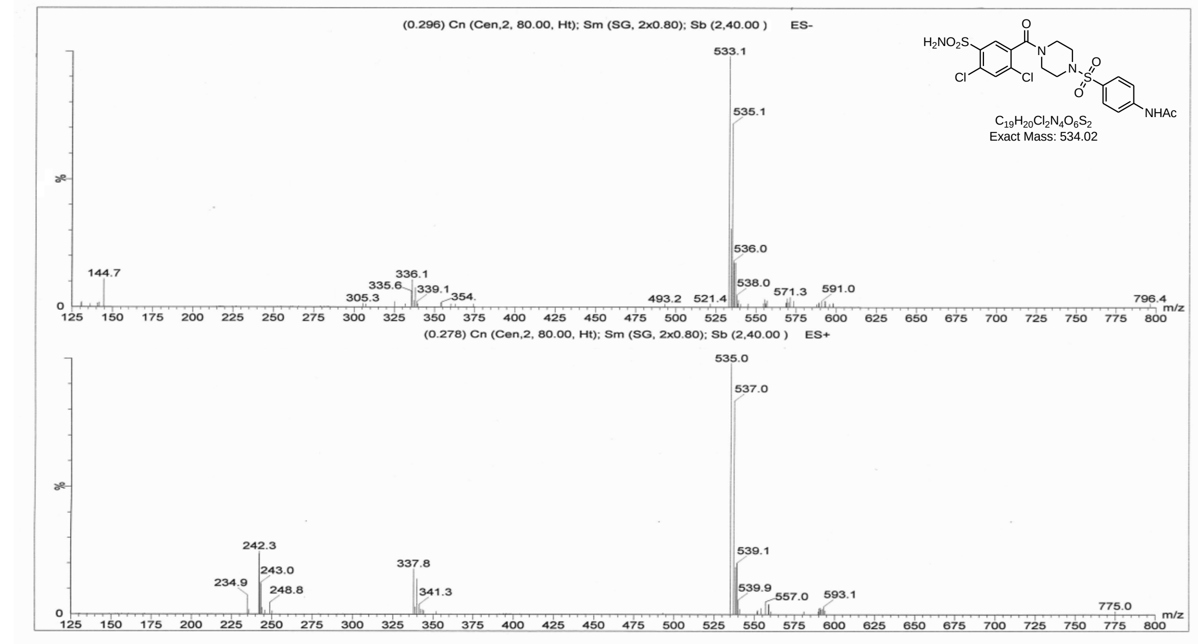

Figure S26: Compound **11** (^{1}H NMR, DMSO-d_6, 700 MHz)

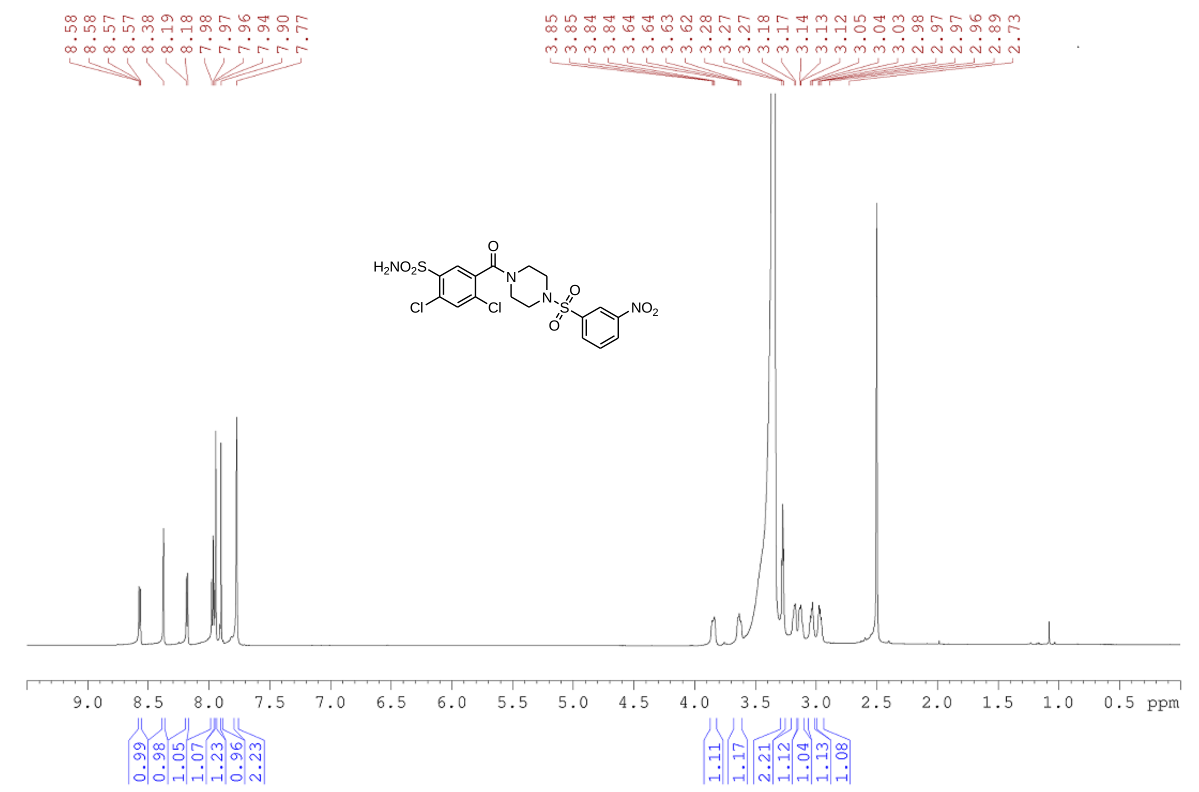

Figure S27: Compound **11** (^{13}C NMR, DMSO-d_6, 176 MHz)

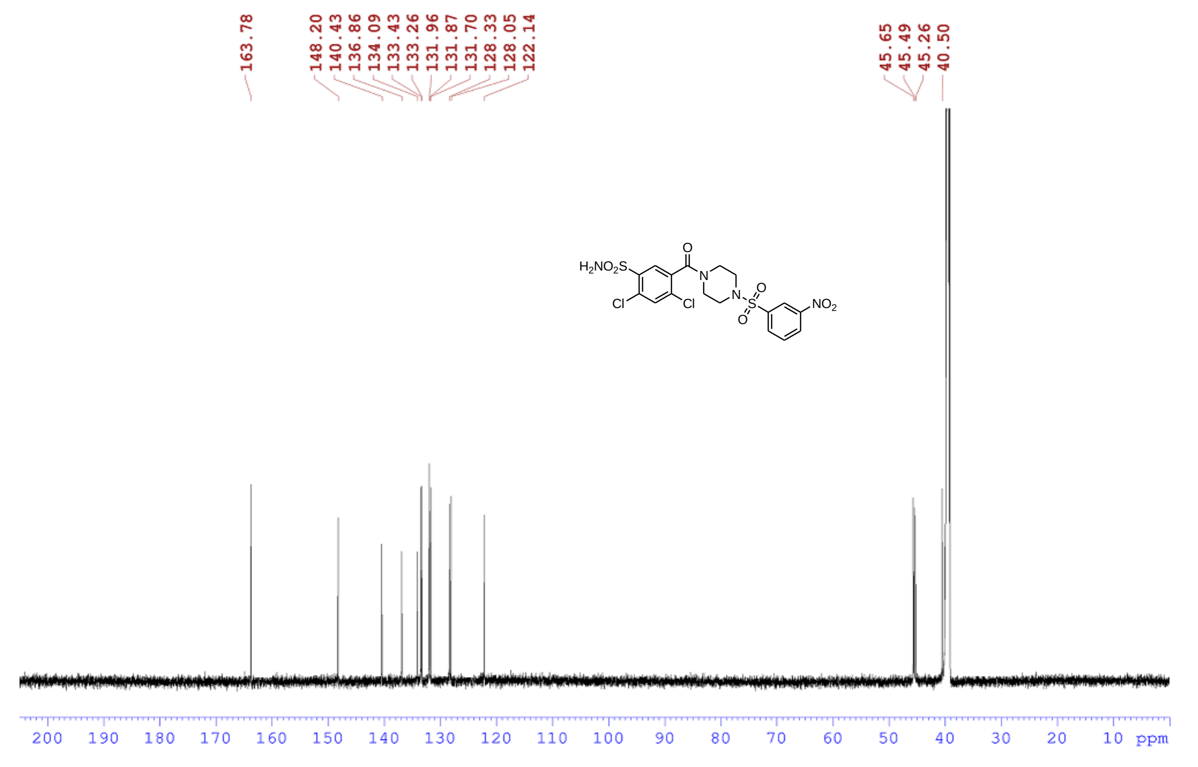

Figure S28: Compound **11** (ESMS)

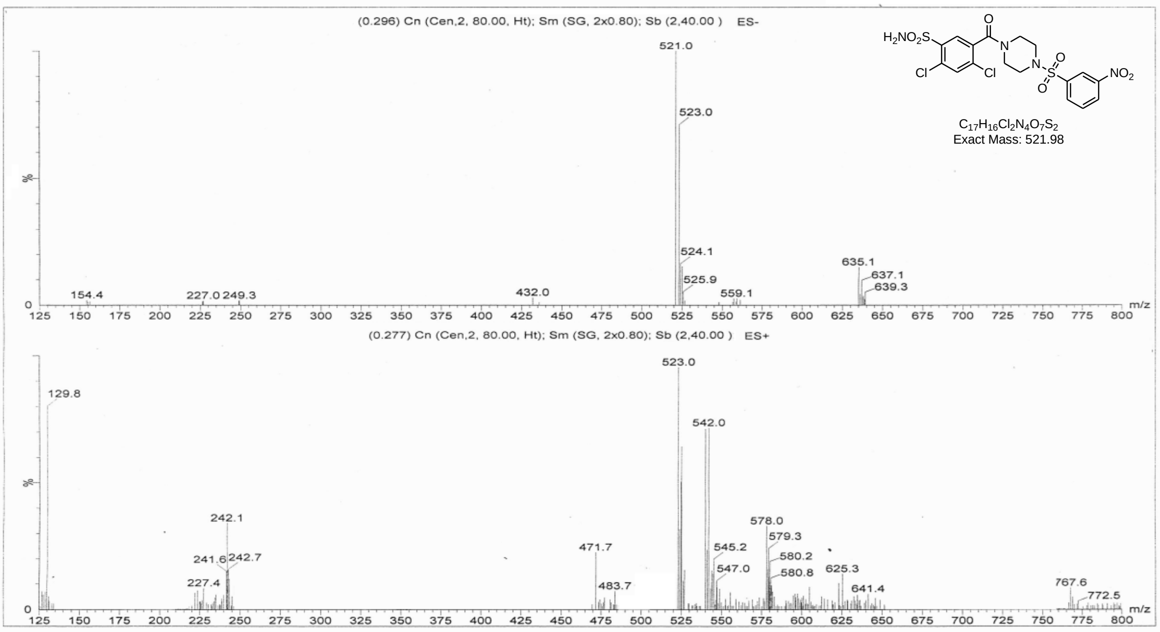

Figure S29: Compound **12** (^{1}H NMR, DMSO-d_6, 700 MHz)

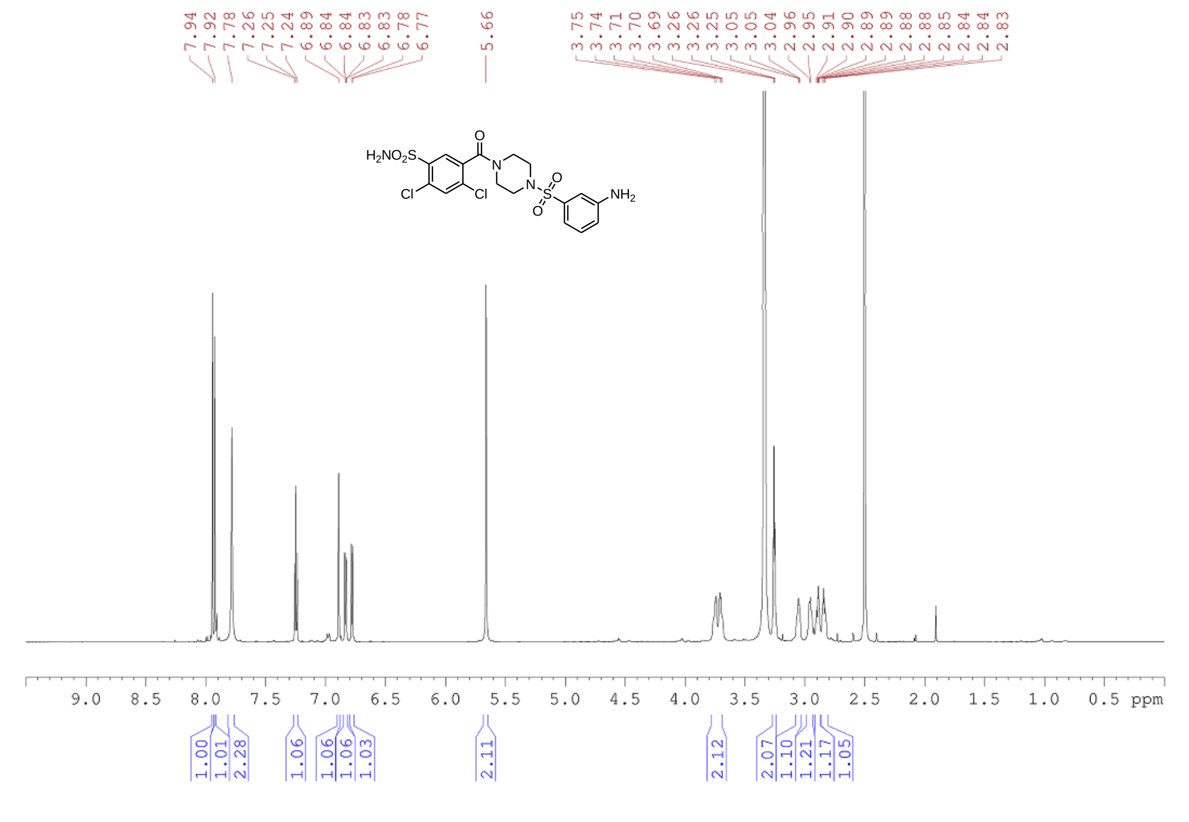

Figure S30: Compound **12** (^{13}C NMR, DMSO-d_6, 176 MHz)

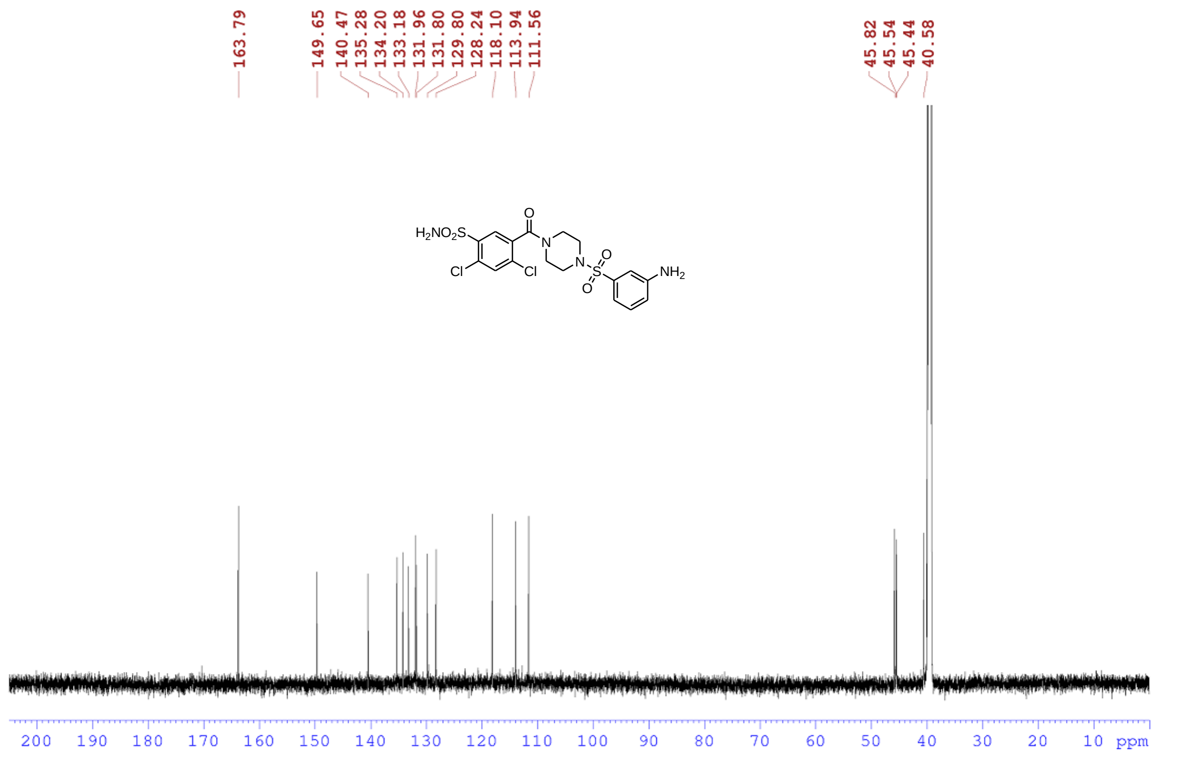

Figure S31: Compound **12** (ESMS)

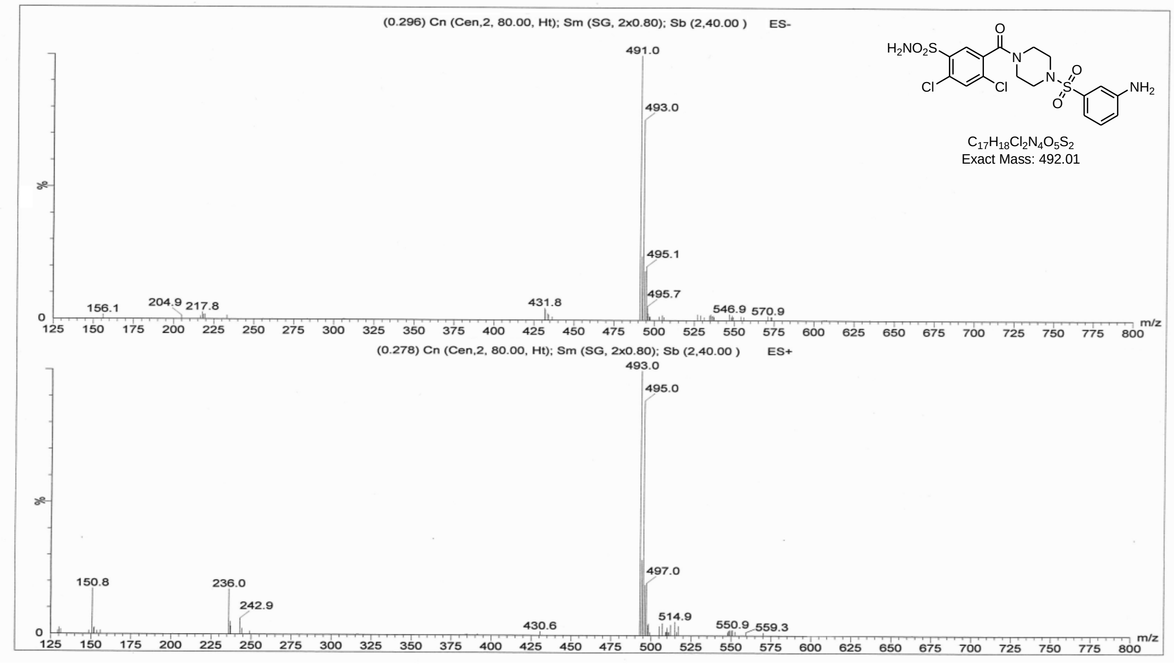

Figure S32: Compound **13** (^{1}H NMR, DMSO-d_6, 700 MHz)

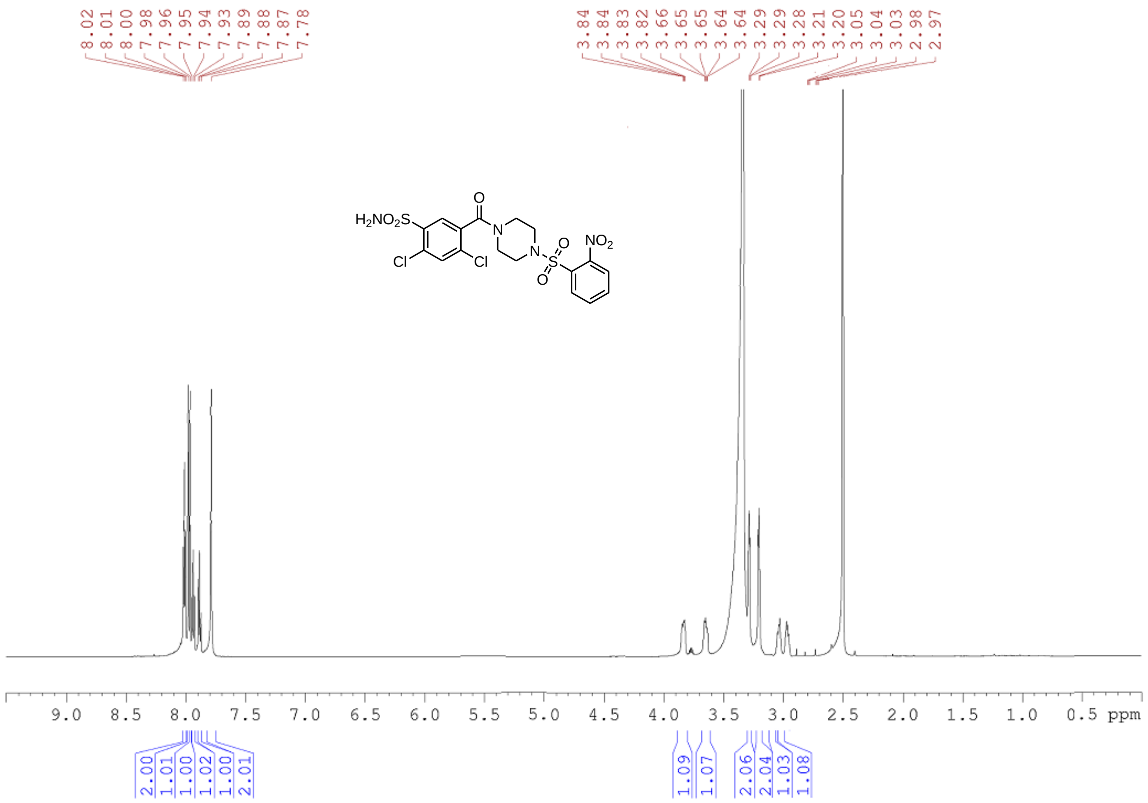

Figure S33: Compound **13** (^{13}C NMR, DMSO-d_6, 176 MHz)

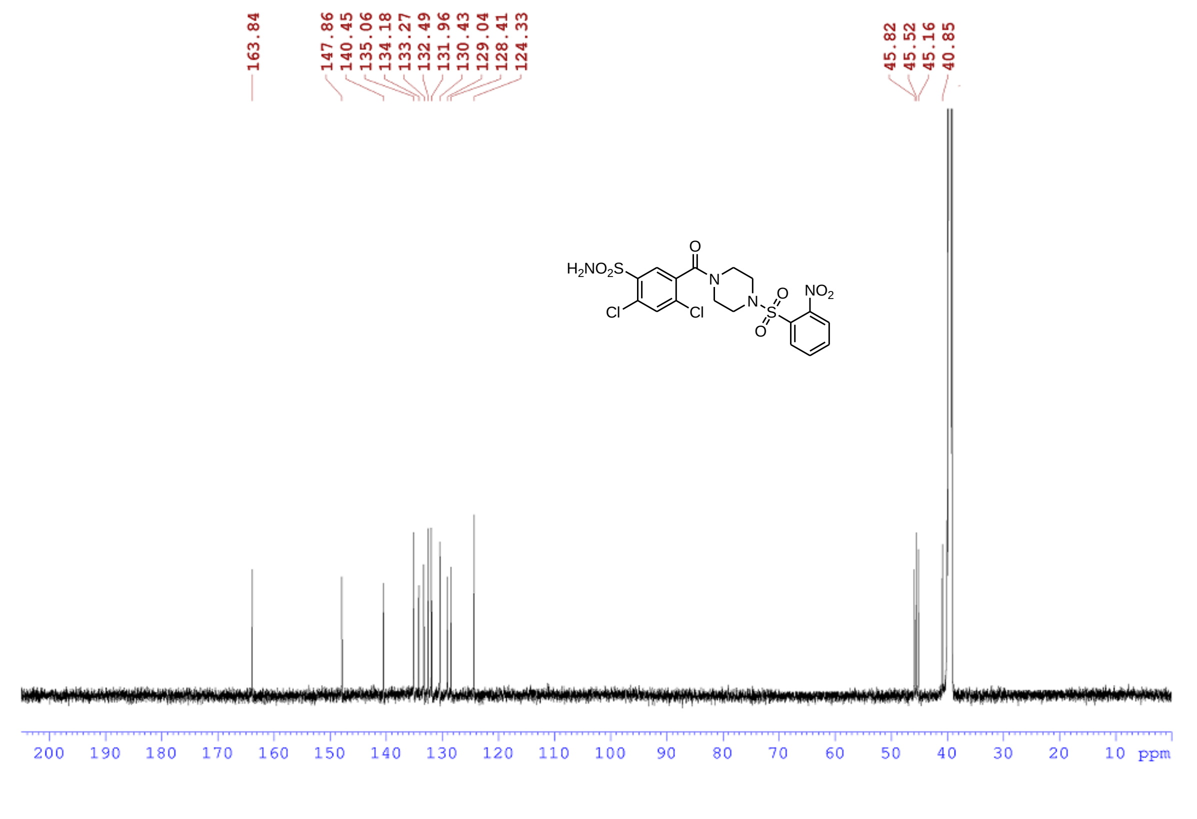

Figure S34: Compound **13** (ESMS)

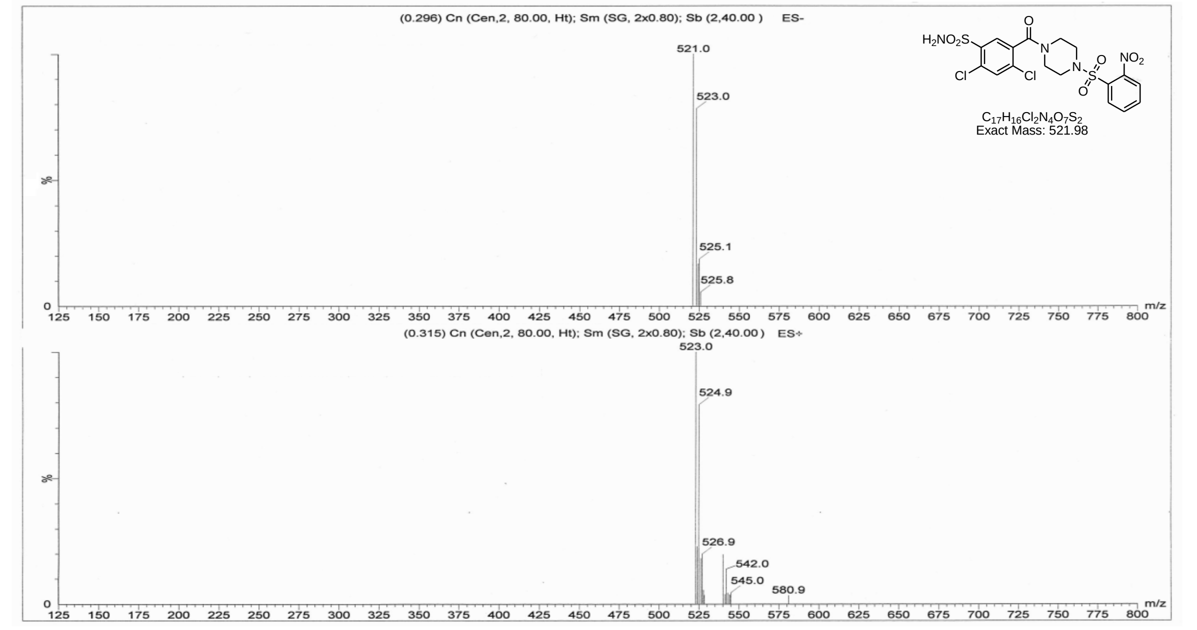

Figure S35: Compound **14** (^{1}H NMR, DMSO-d_6, 700 MHz)

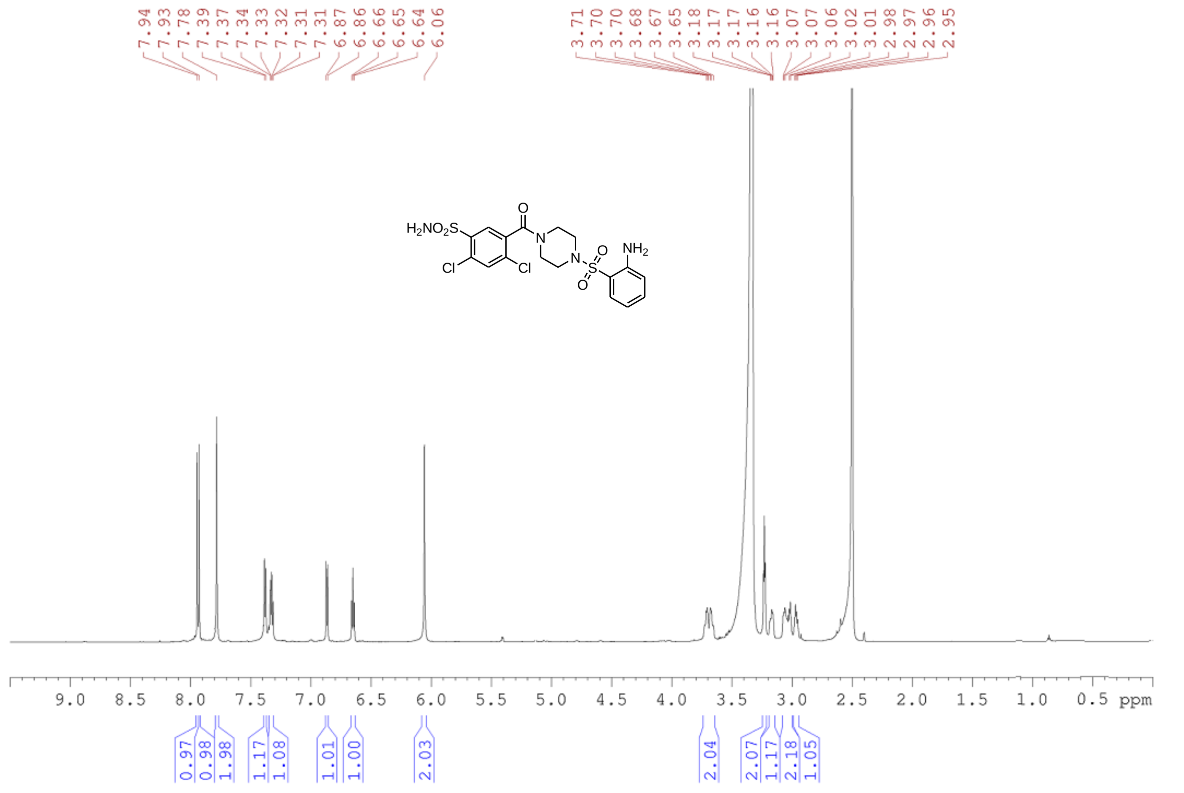

Figure S36: Compound **14** (^{13}C NMR, DMSO-d_6, 176 MHz)

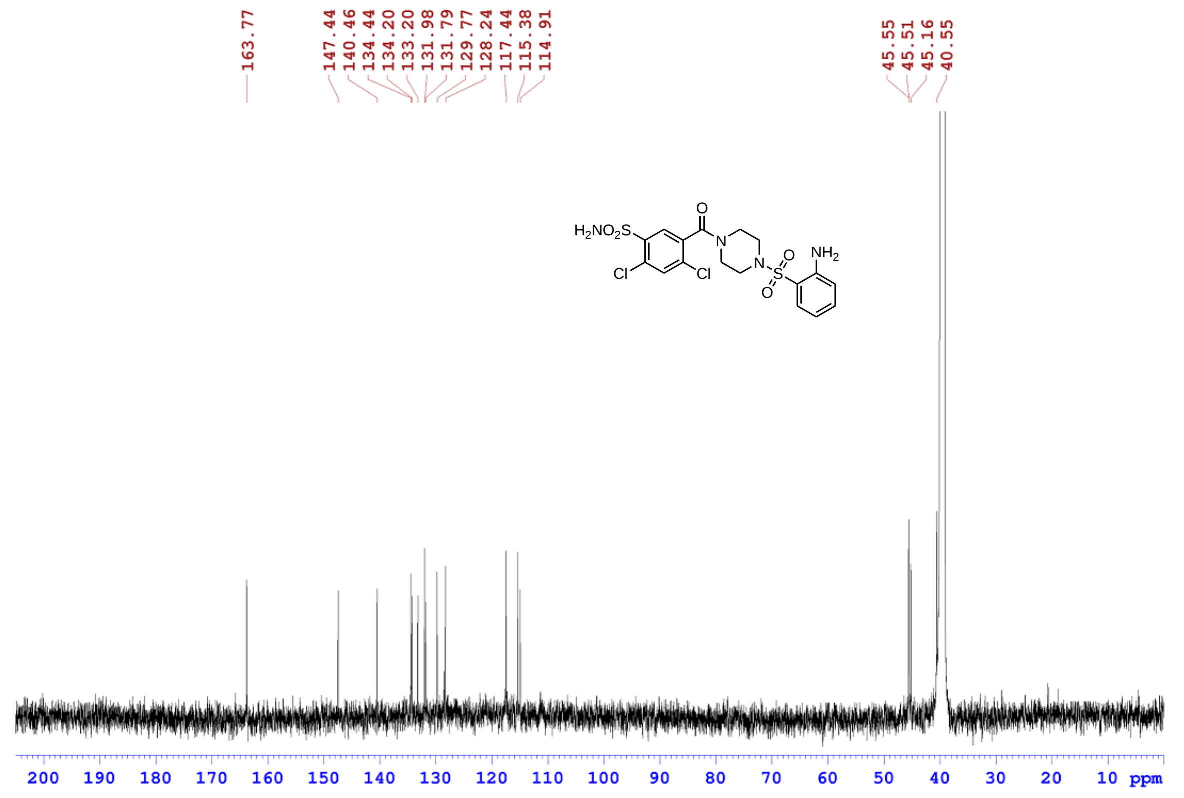

Figure S37: Compound **14** (ESMS)

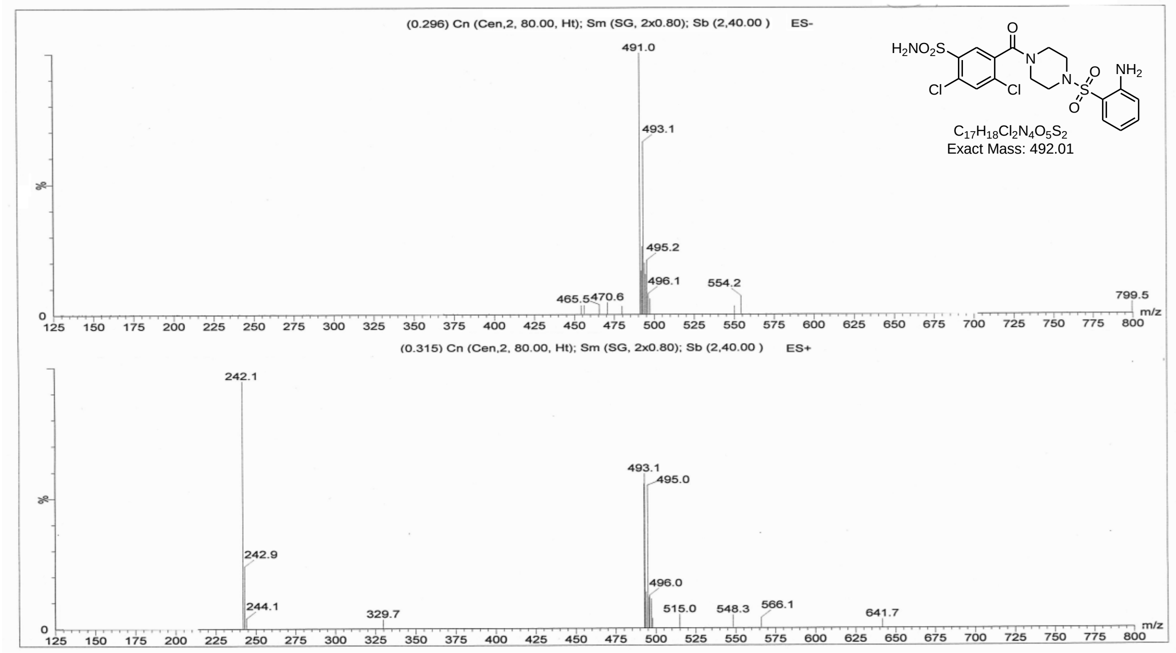

Figure S38: Compound **15** (^{1}H NMR, DMSO-d_6, 700 MHz)

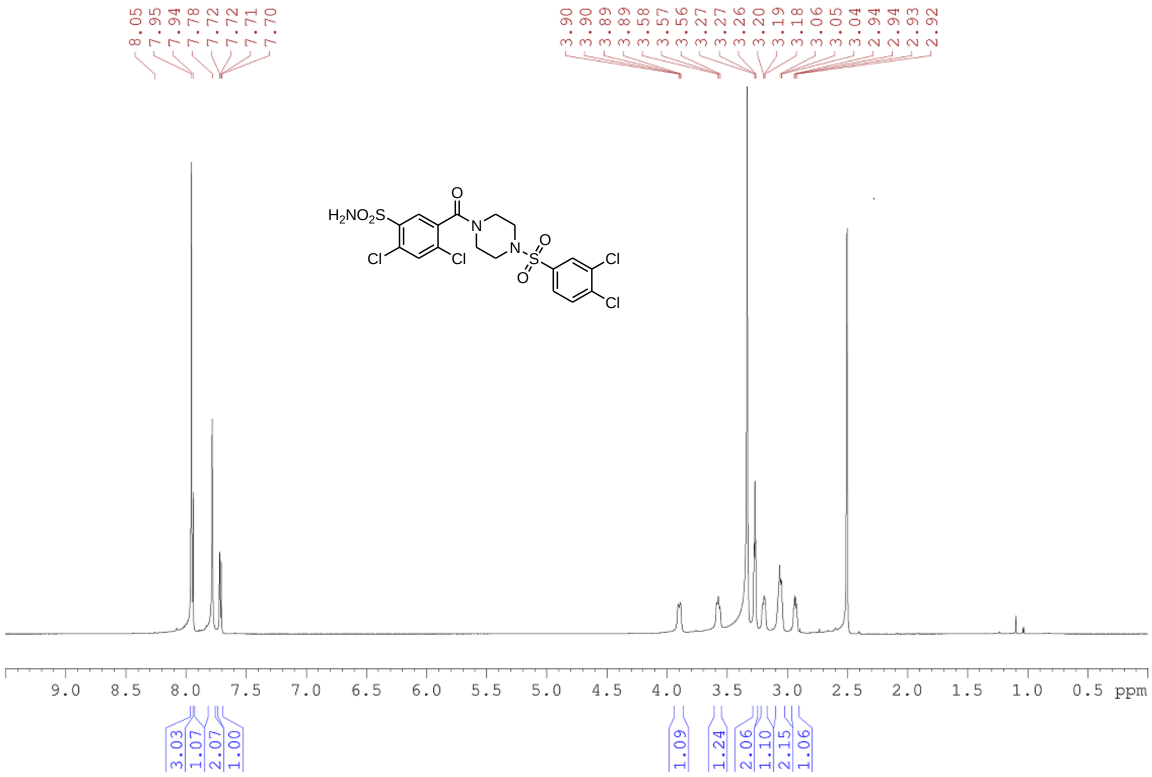

Figure S39: Compound **15** (^{13}C NMR, DMSO-d_6, 176 MHz)

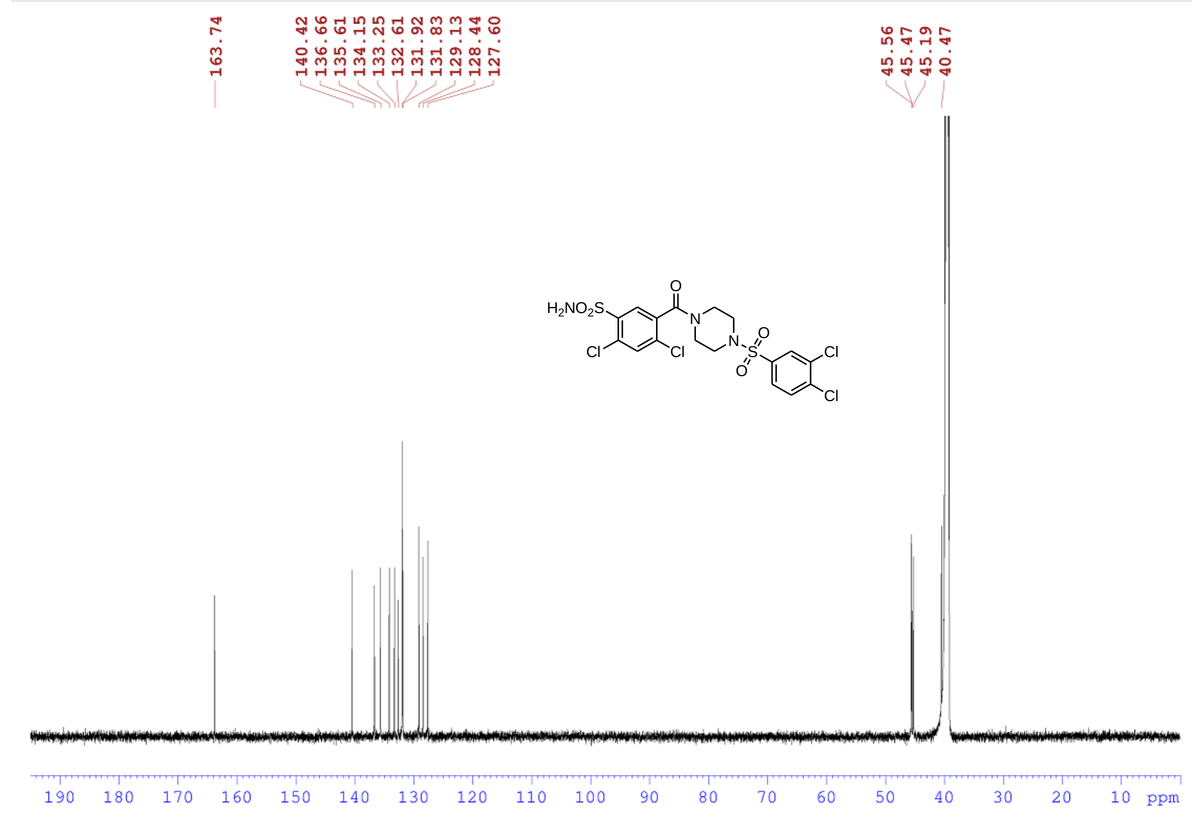

Figure S40: Compound **15** (ESMS)

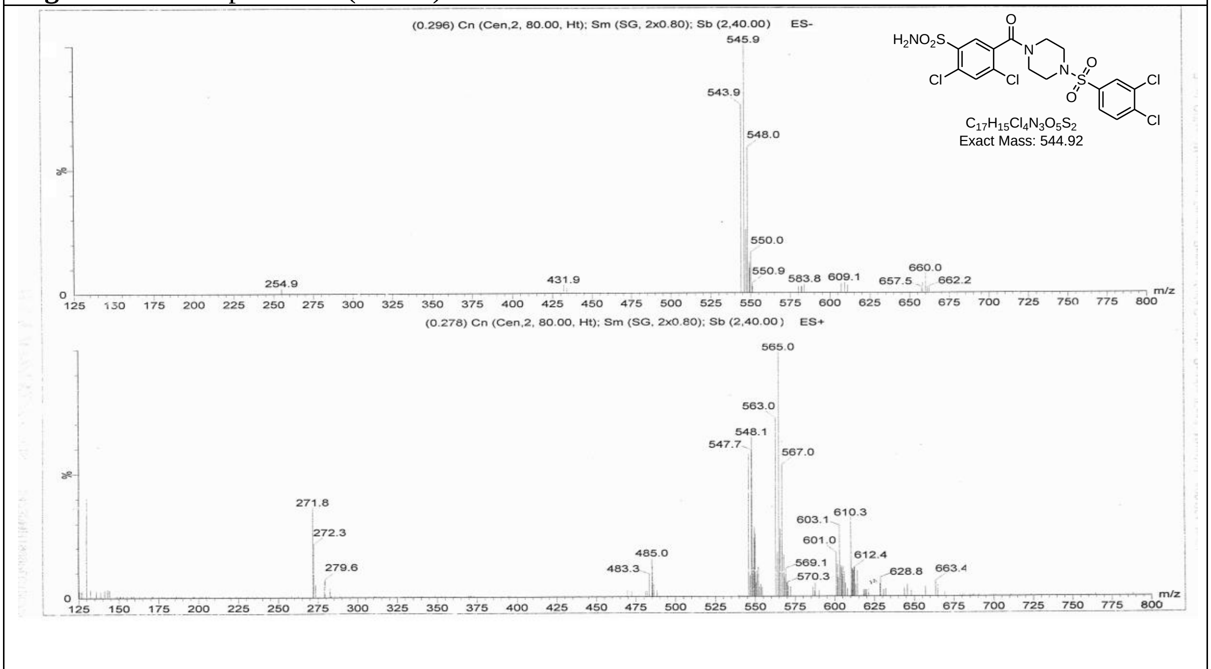

Figure S41: Compound **16** (^{1}H NMR, DMSO-d_6, 700 MHz)

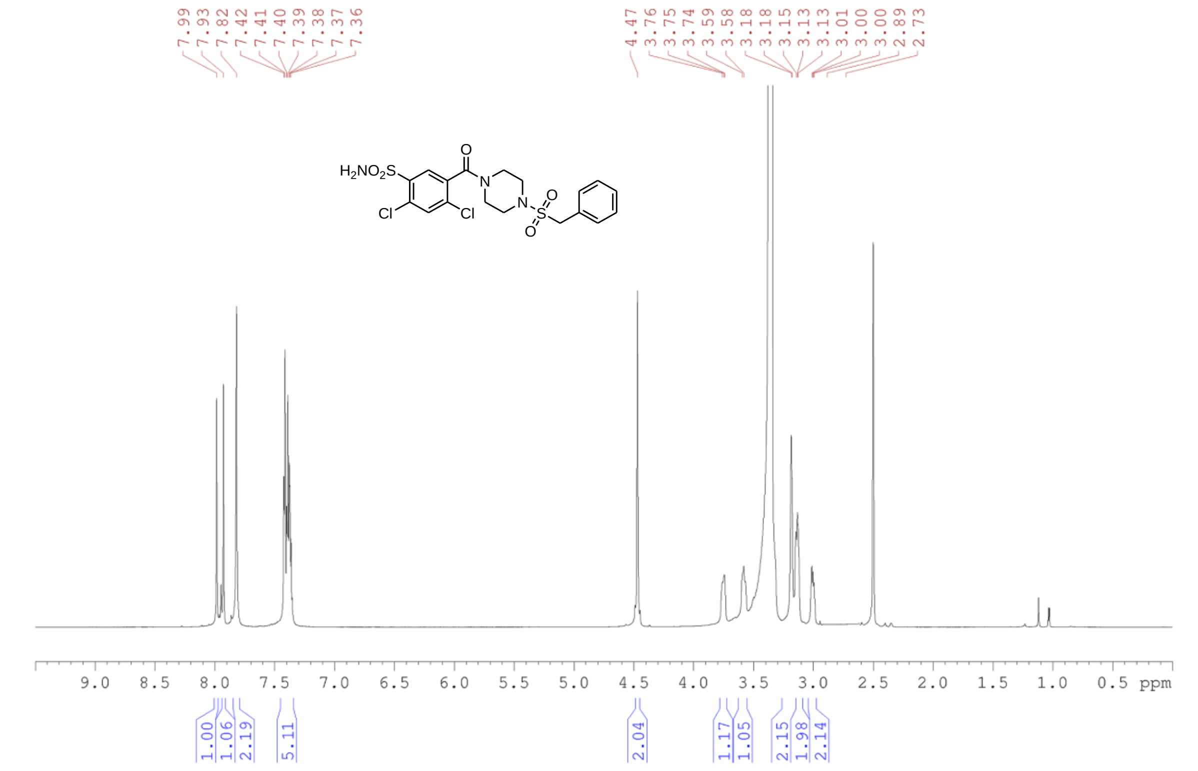

Figure S42: Compound **16** (^{13}C NMR, DMSO-d_6, 176 MHz)

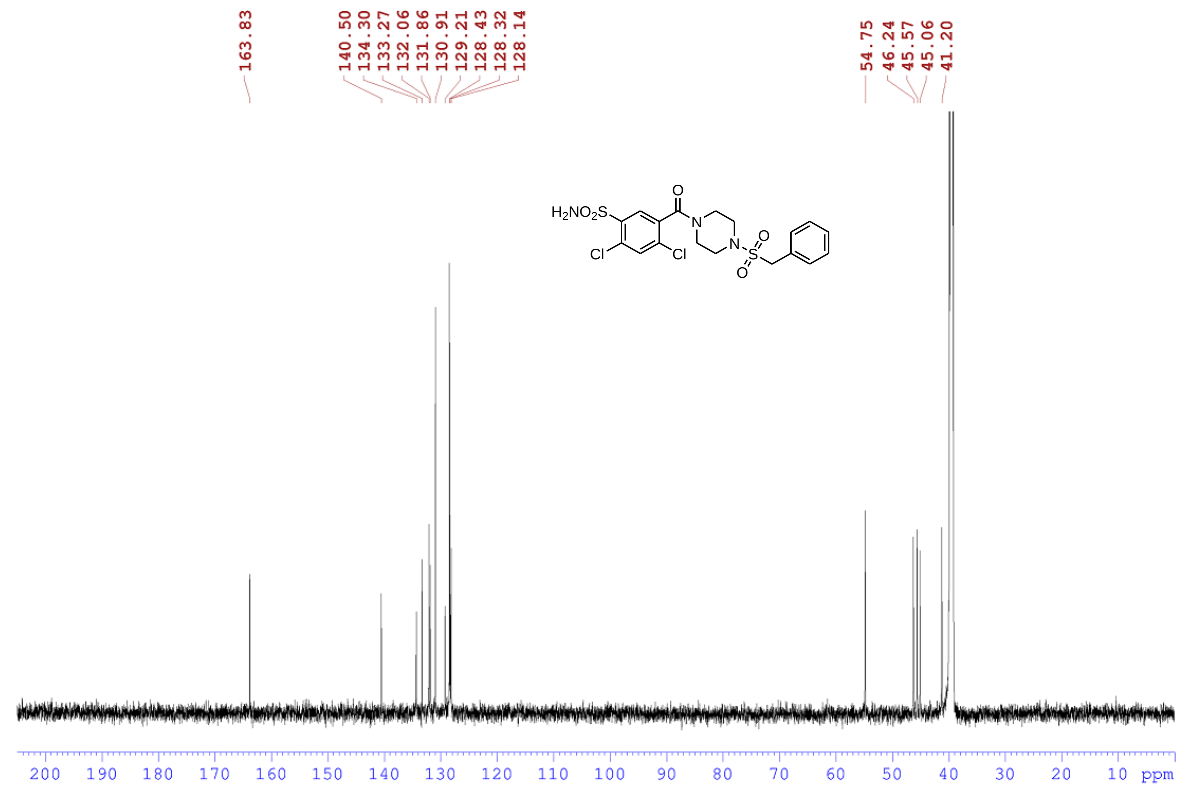

Figure S43: Compound **16** (ESMS)

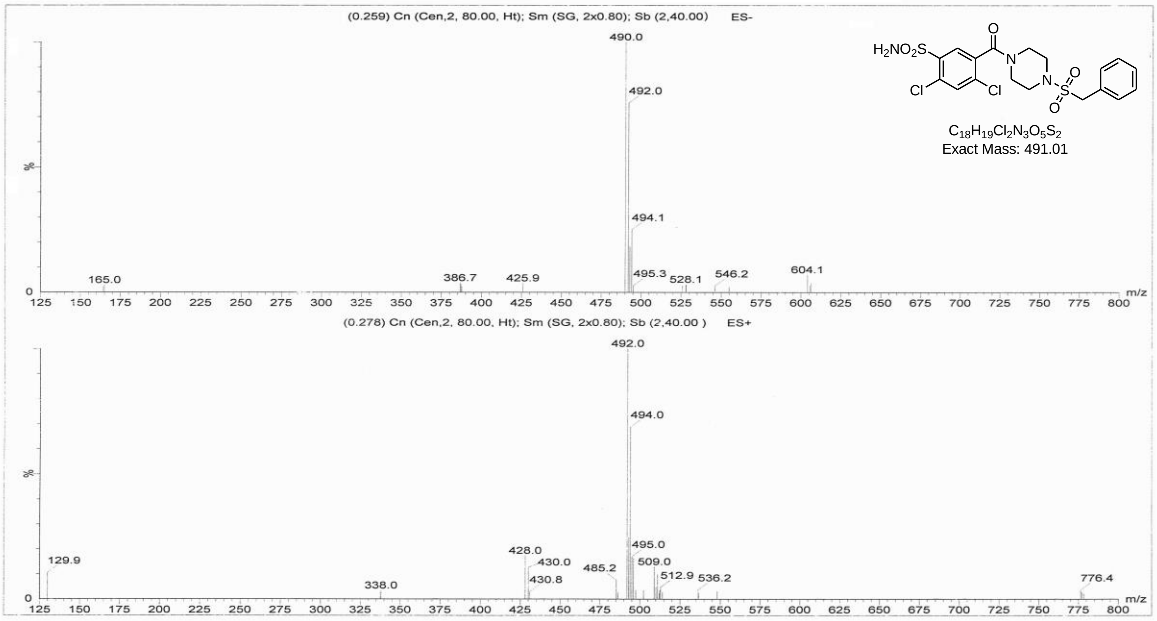

Supplement: Supplementary file 1 — Supporting Information [file CMDC-19-e202400601-s001.pdf]
